# Proteomic and Genomic Analyses of Antimony Resistant *Leishmania infantum* Mutant

**DOI:** 10.1371/journal.pone.0081899

**Published:** 2013-11-27

**Authors:** Marie-Christine Brotherton, Sylvie Bourassa, Philippe Leprohon, Danielle Légaré, Guy G. Poirier, Arnaud Droit, Marc Ouellette

**Affiliations:** 1 Centre de Recherche en Infectiologie du Centre de Recherche du CHU de Québec, Pavillon CHUL, and Département de Microbiologie-Infectiologie et Immunologie, Faculté de Médecine, Université Laval, Québec, Canada; 2 Plate-forme Protéomique du Centre de génomique de Québec, CHU de Québec, Pavillon CHUL, Université Laval, Québec, Canada; Technion-Israel Institute of Technology Haifa 32000 Israel., Israel

## Abstract

**Background:**

Antimonials remain the primary antileishmanial drugs in most developing countries. However, drug resistance to these compounds is increasing and our understanding of resistance mechanisms is partial.

**Methods/Principal Findings:**

In the present study, quantitative proteomics using stable isotope labelling of amino acids in cell culture (SILAC) and genome next generation sequencing were used in order to better characterize *in* *vitro* generated *Leishmania infantum* antimony resistant mutant (Sb2000.1). Using the proteomic method, 58 proteins were found to be differentially regulated in Sb2000.1. The ABC transporter MRPA (ABCC3), a known marker of antimony resistance, was observed for the first time in a proteomic screen. Furthermore, transfection of its gene conferred antimony resistance in wild-type cells. Next generation sequencing revealed aneuploidy for 8 chromosomes in Sb2000.1. Moreover, specific amplified regions derived from chromosomes 17 and 23 were observed in Sb2000.1 and a single nucleotide polymorphism (SNP) was detected in a protein kinase (LinJ.33.1810-E629K).

**Conclusion/Significance:**

Our results suggest that differentially expressed proteins, chromosome number variations (CNVs), specific gene amplification and SNPs are important features of antimony resistance in *Leishmania*.

## Introduction

Protozoan parasites of the genus *Leishmania* cause different clinical manifestations known as leishmaniasis which affect 12 million people worldwide with an estimation of 1.5-2 million new cases each year [1]. Chemotherapy represents the only possible way to fight this disease since no vaccine is available. Pentavalent antimony (SbV) compounds, such as sodium stibogluconate (Pentostam) and meglumine antimoniate (Glucantime), remain the first line of treatment against leishmaniasis especially in developing countries [2]. However, in certain regions, such as Bihar state in India, resistance to SbV is now widespread [3-5]. Other drugs such as paromomycin [6] and the orally administered miltefosine [7] are effective and used nowadays against *Leishmania*. Nonetheless, despite its high cost limiting its use in developing countries [8], liposomal AmB is now considered as the best drug available against visceral leishmaniasis, the most severe form of the disease [9,10]. 

The mechanisms of resistance to antimony-based drugs were extensively studied in *Leishmania*. A decrease in the reduction rate from SbV to SbIII, the active form against the parasite, was observed in Pentostam-resistant amastigotes [11]. A decrease in the expression of the aquaglyceroporin AQP1, responsible for the uptake of SbIII, was also observed in *in vitro* resistant mutants [12,13] and unresponsive clinical strains [14,15]. The gene coding for the ABC transporter MRPA (ABCC3), involved in the sequestration of SbIII once conjugated with thiols, was amplified on an extrachromosomal circular amplicon in *in vitro* generated mutants [16-20] and clinical unresponsive strains [21,22]. An increase in the level of intracellular thiols, such as cysteine, glutathione and trypanothione, was observed in numerous laboratory-selected resistant strains [18,19,23-27] as well as in unresponsive clinical strains [14,21,22,28,29]. Other proteins were also linked with the antimony resistance phenotype (reviewed in [30]).

Quantitative proteomics and whole genome sequencing methodologies are now emerging as powerful approaches to study drug resistance in microorganisms. In the parasite *Leishmania*, proteomic studies have already revealed some resistance mechanisms to antimony [31-35]. Similarly, whole genome sequencing has proven useful to study antimony resistance in clinical *L. donovani* isolates [36] and in *in vitro* resistant *L. major* mutants [37]. In the present study, we used a combination of quantitative proteomics using SILAC and whole genome sequencing to characterize *in vitro* selected SbIII resistant mutant.

## Methods

### Cell culture

*L. infantum* (MHOM/MA/67/ITMAP-263) wild-type (WT) and the *in vitro* generated resistant mutant Sb2000.1 [20,38-40], which is resistant to 2000 µM of SbIII, were described previously. Promastigotes were grown in RPMI-1640 medium for SILAC (minus L-lysine and L-arginine) (Cambridge Isotope Laboratories) supplemented with 75 µM adenosine (Sigma), 28 mM HEPES (Sigma), 40 µM biotin (Sigma), 1% penicillin-streptomycin (Wisent), 5 mg/L hemin (MP Biomedicals), 10 µM biopterin and 10% heat-inactivated dialysed foetal bovine serum (Cambridge Isotope Laboratories). *L. infantum* WT light medium (normal isotopic abundance) was also supplemented with 242.26 mg/L L-arginine (Sigma) and 50.03 mg/L L-lysine, while 253.68 mg/L ^13^C_6_-^15^N_4_-L-arginine and 62.21 mg/L ^13^C_6_-^15^N_2_-L-lysine were added to the resistant mutant heavy medium. Mutant cells were grown in this medium for at least 7 passages to ensure a minimum of 99% of isotope incorporation. Cells from each WT and mutant cultures were counted using a haemocytometer and the resistant strain was then mixed with WT cells in a 1:1 ratio. The SILAC labeling and subsequent mass spectrometry quantification of labeled peptides was done once.

### Sample preparation

The membrane-enriched (ME) fraction was obtained by sonication, ultracentrifugation and further purification by Free flow zone electrophoresis (ZE-FFE) as described previously [41]. The cytosolic protein extraction was performed in 2D lysis buffer as described previously [42]. Proteins were then quantified using the 2D Quant kit (GE Healthcare). 

### Sodium dodecyl sulfate (SDS)-PAGE (1DE)

Protein samples (30 µg) were mixed with 4x premixed protein sample buffer (BioRad) and β-mercaptoethanol (5% final concentration, Sigma), and heated at 95°C for 5 min. Protein mixtures were then loaded on Precast Criterion XT Bis-Tris gradient gels (4-12% polyacrylamide, BioRad) and the SDS-PAGE separation was performed on a Criterion™ gel electrophoresis cell (BioRad) using a PowerPac 200 BioRad power supply set at 200 V for 50 min. For staining, gels were fixed in a solution of 50% methanol: 7.5% acetic acid for 1 h then incubated overnight with SYPRO Ruby Protein Gel stain (BioRad). The destaining step was performed for 30 min in a solution of 15% methanol: 7.5% acetic acid. Gel images were captured on a PerkinElmer ProExpress Proteomic Imaging system. Each sample lane from the SDS-PAGE gels was cut in 40 fractions (24 fractions above 50 kDa and 16 fractions below 50 kDa) with disposable blade (MEE-1x5) mounted on a One Touch GridCutter (Gel Company Inc.).

### Protein in-gel digestion

Bands of interest were extracted from SDS-PAGE gels, placed in 96-well plates and washed extensively with HPLC water. Tryptic digestion was performed on a MassPrep liquid handling robot (Waters) according to the manufacturer’s specifications and to the protocol of Shevchenko et al [43] with modifications [44]. Briefly, proteins were reduced with 10 mM DTT and alkylated with 55 mM iodoacetamide. Trypsin digestion was performed using 126 nM of modified porcine trypsin (Sequencing grade, Promega) at 58 °C for 1 h. Digestion products were then extracted using 1% formic acid and 2% acetonitrile followed by 1% formic acid and 50% acetonitrile. The recovered protein extracts were pooled, vacuum centrifuge dried and resuspended into 10 µL of 0.1% formic acid. Aliquots of 2 (for cytosolic fractions) or 5 (for ME fractions) µL were analyzed by mass spectrometry.

### Mass spectrometry for ME fraction

SILAC experiments for ME fraction were performed on a QSTAR XL QqTOF mass spectrometer equipped with a nanospray II ion source (ABSciex) coupled to an Agilent 1100 HPLC. Five microliters of each protein sample were injected by the Agilent 1100 autosampler onto a 0.075 mm (internal diameter) self-packed IntegraFrit column (New Objective) packed with an isopropanol slurry of 5 µm Jupiter C18 (Phenomenex) stationary phase using a pressure vessel set at 700 psi. The length of the column was 12 cm. Samples were run using a 75 min gradient from 10-40% solvent B (solvent A: 0.1% formic acid in water; solvent B: 0.1% formic acid in acetonitrile) at a flow rate of 250 nL/min. An information-dependant acquisition (IDA) method was set up with the MS survey range set between 400 amu and 1600 amu (1 s) followed by dependent MS/MS scans with a mass range set between 100 and 1600 amu (3 s) of the 3 most intense ions with the enhanced all mode activated. Dynamic exclusion was set for a period of 15 s and a tolerance of 100 ppm. 

### Mass spectrometry for cytosolic fraction

SILAC experim*ents for cytosolic protein fraction were performed on a TripleTOF 5600 mass spectrometer equipped with a nanospray III ion source (ABSciex) coupled to an Agilent 1200 HPLC. Two microliter samples were injected by the Agilent 1200 autosampler onto a 0.075 mm (internal diameter) self-packed PicoFrit column (New Objective) packed with an isopropanol slurry of 5 µm Jupiter C18 (Phenomenex) stationary phase using a pressure vessel set at 700 psi. The length of the column was 15 cm. Samples were run using a 65 min gradient from 5-35% solvent B (solvent A: 0.1% formic acid in water; solvent B: 0.1% formic acid in acetonitrile) at a flow rate of 300 nL/min. Data were acquired using an ion spray voltage of 2.4 kV, curtain gas of 30 psi, nebulizer gas of 8 psi and an interface heater temperature of 125 °C. An information-dependant acquisition (IDA) method was set up with the MS survey range set between 400 amu and 1250 amu (250 ms) followed by dependent MS/MS scans with a mass range set between 100 and 1800 amu (50 ms) of the 20 most intense ions in the high sensitivity mode with a 2+ to 5+ charge state. Dynamic exclusion was set for a period of 3 s and a tolerance of 100 ppm. 

### Interpretation of tandem mass spectra and protein identification

Raw data files (n=40 for each sample) were submitted for simultaneous searches using the Protein Pilot version 4 software (ABSciex) utilizing the Paragon and Progroup algorithms [45]. The Protein Pilot program was set up to search the *L. infantum* proteins in the TriTrypDB LeishPEP database (http://tritrypdb.org/common/downloads/release-4.0/Linfantum/fasta/LinfantumAnnotatedProteins_TriTrypDB-4.0.fasta) with carbamidomethyl (C) as a fixed modification and standard SILAC (Lys +8, Arg +10) settings for QSTAR or TripleTof 5600 instruments. Proteins for which at least two fully trypsin-digested light (L) and heavy (H) peptides were detected at > 99% confidence and quantitative p-value lower than 0.05 were used for subsequent comparative quantitative analysis.

### Whole genome sequencing

Genomic DNAs were prepared from mid-log phase cultures of clonal populations of *L. infantum* 263 WT and Sb2000.1 as described previously [46]. Their sequences were determined by Illumina MiSeq 150-nucleotides paired-end reads which assembled into 3197 and 2920 contigs of at least 500 nucleotides for *L. infantum* 263 WT and Sb2000.1, respectively. Sequence reads from each strain were aligned to the reference genome *L. infantum* JPCM5 [47] available at TriTrypDB (version 4.0) [48] using the software bwa (bwa aln, version 0.5.9) with default parameters [49]. The maximum number of mismatches was 4, the seed length was 32 and 2 mismatches were allowed within the seed. The detection of single nucleotide polymorphisms (SNPs) was performed using SAMtools (version 0.1.18), bcftools (distributed with SAMtools) and vcfutils.pl (distributed with SAMtools) [50], with a minimum of three reads to call a potential variation prior to further analysis. Sequencing data are available at the EMBL-EBI European Nucleotide Archive (http://www.ebi.ac.uk/ena) under study accession number ERP001815 with samples ERS179382 and ERS176090 corresponding to *L. infantum* 263 WT and Sb2000.1, respectively. Several python (version 2.4.3) and bash (version 3.2) scripts were created to further analyze the data. The quality assessment software SAMStat (v1.08) was used to generate quality reports [51]. Putative SNPs detected by whole genome sequencing were verified by conventional PCR amplification and DNA sequencing. Growth curves were obtained by measuring absorbance at 600 nm of 72-hour cultures with different drug concentrations using an automated microplate reader.

### DNA constructs

The gene LinJ.33.1810 was amplified from genomic DNA derived from *L. infantum* 263 WT. The PCR fragment was purified and digested with both XbaI and HindIII before being cloned into the *Leishmania* expression vector *pSP72αNEOα* [52] digested with the same enzymes. The integrity of the cloned open reading frame was confirmed by conventional sequencing before being used for transfection.

## Results

### Proteomic analysis of the antimony resistant mutant

The *L. infantum* Sb2000.1 mutant resistant to SbIII was grown in the presence of ^13^C_6_-^15^N_4_-L-arginine and ^13^C_6_-^15^N_2_-L-lysine while the parental *L. infantum* WT strain was maintained in medium containing normal isotopic abundance amino acids. Cells were counted and mutant was mixed with WT cells in a 1:1 ratio. ME fraction and cytosolic proteins were independently extracted from mixed populations and subjected separately to SDS-PAGE separation. Sample lines were further fractionated into 40 pieces of gel, proteins were gel extracted, trypsin digested and peptides were identified and quantified by mass spectrometry and isotopic quantification. Only significant protein identifications with difference in their expression level greater than 1.2-fold between mutant and WT were considered. Thus, 60 differentially expressed proteins were observed in cytosolic or ME fractions of Sb2000.1 when compared to the WT strain (Table 1). Among them, 42 (70%) were up-regulated and 18 (30%) were down-regulated in Sb2000.1. Two proteins were identified independently in both fractions, but varied in the same way (LinJ.21.0310 and LinJ.24.1700) (Table 1). All the identified cytosolic proteins were predicted to be devoid of transmembrane domains (TMDs) according to the TMHMM v2.0 algorithm, whereas 40% of the proteins differentially expressed in ME fractions encoded at least one TMD (Table 1). The latter result is consistent with a previous study using the same method to obtain ME fractions from promastigote parasites [41].

**Table 1:** MS/MS identifications of differentially expressed proteins in Sb2000.1 compared to WT in SILAC experiments*^a^*.

**Systematic IDs**	**Putative protein name**	**Unique peptides** ^*b*^ **(% seq. coverage**)		**Ratio Sb2000.1/WT**		**P-value**		**Error Factor**		**TMDs** ^*c*^		**Fraction**	
**Transport**													
LinJ.05.1140	vacuolar ATPase subunit-like protein	2 (5.88)		1,24		0,0583		1,26		0		M	
LinJ.10.0400	FT1 folate/biopterin transporter, putative	3 (5.97)		-2,22		0,0450		2,15		11		M	
LinJ.10.0420	FT5 folate/biopterin transporter, putative	6 (6.99)		-1,65		0,0618		1,76		11		M	
LinJ.11.1050	pretranslocation protein, alpha subunit, putative, SEC61-like (pretranslocation process) protein, putative	2 (2.26)		1,46		0,0595		1,50		9		M	
LinJ.23.0290	MRPA (ABCC3) ABC-thiol transporter	13 (9.37)		3,58		0,0002		1,76		8		M	
LinJ.31.1240	vacuolar-type proton translocating pyrophosphatase 1, putative	9 (10.86)		-1,36		0,0374		1,33		14		M	
LinJ.32.2150	cop-coated vesicle membrane protein p24 precursor, putative,ER--golgi transport protein p24, putative	3 (24.38)		1,63		0,0267		1,42		1		M	
LinJ.35.1840	COP-coated vesicle membrane protein gp25L precursor, putative,ER--golgi transport protein gp25L, putative	4 (18.69)		1,42		0,0160		1,25		2		M	
**Surface**													
LinJ.10.0500	GP63, leishmanolysin,metallo-peptidase, Clan MA(M), Family M8	23 (26.04)		-1,91		1,257E-07		1,17		1		M	
LinJ.30.0920	surface protein amastin, putative	5 (26.26)		-1,73		0,0196		1,52		4		M	
**Cytoskeleton**													
LinJ.16.1550	kinesin, putative	4 (65.24)		2,75		0,0010		1,28		0		M	
LinJ.23.0720	kinesin, putative	3 (3.51)		1,67		0,0013		1,19		0		M	
**Metabolism**													
LinJ.01.0510	long chain fatty acid CoA ligase, putative	4 (6.45)		1,47		0,0078		1,26		0		M	
LinJ.06.1340	protoporphyrinogen oxidase-like protein	2 (14.29)		-2,95		0,0084		1,74		1		M	
LinJ.10.0310	isocitrate dehydrogenase [NADP], mitochondrial precursor, putative	3 (6.21)		1,27		0,0722		1,31		0		C	
LinJ.11.1000	pyruvate phosphate dikinase, putative	2 (1.20)		1,38		0,0028		1,16		0		M	
LinJ.11.1100	sterol 14-alpha-demethylase	6 (10.63)		1,45		0,0085		1,28		0		M	
LinJ.13.0960	NADH-cytochrome B5 reductase, putative	6 (17.86)		1,47		0,0255		1,36		1		M	
LinJ.15.1140	tryparedoxin peroxidase	12 (42.71)		1,38		0,0623		1,44		0		C	
LinJ.21.0310	hexokinase, putative	2 (6.16)		1,40		0,0182		1,26		0		C	
LinJ.21.0310	hexokinase, putative	10 (16.35)		1,53		0,0034		1,26		0		M	
LinJ.24.0310	fumarate hydratase, putative	2 (4.55)		1,25		0,0766		1,31		0		C	
LinJ.24.1700	succinate dehydrogenase flavoprotein, putative	2 (4.12)		1,20		0,0701		1,22		0		C	
LinJ.24.1700	succinate dehydrogenase flavoprotein, putative	8 (10.05)		1,37		0,0771		1,44		1		M	
LinJ.30.2590	formate--tetrahydrofolate ligase	1 (2.25)		1,57		0,0408		1,50		0		C	
LinJ.30.2920	aldehyde dehydrogenase, putative	3 (3.59)		1,55		0,0089		1,25		0		M	
LinJ.30.3000	glyceraldehyde 3-phosphate dehydrogenase, glycosomal	2 (3.88)		1,39		0,0778		1,47		0		M	
LinJ.32.3510	GCVL-2 dihydrolipoamide dehydrogenase, putative	5 (5.25)		1,23		0,0017		1,11		0		M	
LinJ.36.3630	2-oxoglutarate dehydrogenase E1 component, putative	3 (3.56)		1,38		0,0840		1,46		0		M	
LinJ.36.6960	2,3-bisphosphoglycerate-independent phosphoglycerate mutase	10 (21.16)		1,26		0,0471		1,25		0		C	
**Protein folding**													
LinJ.27.2350	heat shock protein DNAJ, putative		5 (16.92)		-1,25		0,0708		1,28		0		C	
LinJ.28.3060	heat-shock protein hsp70, putative	5 (6.79)		1,43		0,0018		1,17		0		M	
LinJ.30.2540	heat shock 70-related protein 1, mitochondrial precursor, putative	3 (4.85)		1,25		0,0373		1,23		0		M	
LinJ.36.2140	chaperonin Hsp60, mitochondrial precursor	40 (52.49)		1,32		0,0008		1,14		0		C	
**Proteolysis**													
LinJ.14.0920	calpain-like cysteine peptidase, putative,cysteine peptidase, Clan CA, family C2, putative	2 (17.39)		-1,44		0,0570		1,48		0		C	
LinJ.20.1320	calpain-like cysteine peptidase, putative,calpain-like cysteine peptidase, Clan CA, family C2	6 (52.32)		-2,55		0,0023		1,64		0		M	
LinJ.27.0500	calpain-like cysteine peptidase, putative,cysteine peptidase, Clan CA, family C2, putative	2 (6.17)		1,72		0,0060		1,28		0		M	
LinJ.36.1730	proteasome beta 5 subunit, putative	3 (11.92)		1,31		0,0345		1,27		0		C	
**Transcription, Translation**													
LinJ.17.0110	elongation factor 1-alpha		11 (21.87)		1,26		0,0003		1,11		0		M	
LinJ.29.1920	40S ribosomal protein S15A, putative	7 (25.38)		1,24		0,0576		1,25		0		M	
LinJ.29.2580	60S ribosomal protein L13, putative	4 (12.27)		-1,26		0,0515		1,26		0		M	
LinJ.30.3650	40S ribosomal protein S14	4 (20.14)		-1,59		0,0021		1,20		0		C	
LinJ.36.0020	histone H4	6 (34.00)		1,41		0,0569		1,43		0		M	
**Others**													
LinJ.20.1350	SMP-1 small myristoylated protein-1, putative	10 (19.85)		1,28		0,0941		1,42		0		M	
LinJ.28.2430	glycosomal membrane protein, putative	3 (11.71)		1,62		0,0007		1,20		0		M	
LinJ.36.1420	Transitional endoplasmic reticulum ATPase, putative,valosin-containing protein homolog	8 (14.78)		-1,26		0,0774		1,30		0		C	
**Hypothetical**													
LinJ.06.0640	hypothetical protein, conserved	4 (5.05)		-1,29		0,0242		1,22		0		C	
LinJ.08.1010	hypothetical protein, conserved	4 (3.01)		1,34		0,0688		1,42		0		M	
LinJ.14.0460	hypothetical protein, conserved	2 (12.11)		-1,40		0,0808		1,51		0		C	
LinJ.17.0010	hypothetical protein, conserved	8 (7.62)		1,26		0,0252		1,21		0		M	
LinJ.24.2250	hypothetical protein, conserved	3 (7.48)		1,47		0,0123		1,25		0		M	
LinJ.26.1020	hypothetical protein, conserved	3 (4.19)		1,32		0,0125		1,19		1		M	
LinJ.26.1960	hypothetical protein, conserved	4 (4.48)		1,29		0,0030		1,15		0		M	
LinJ.27.0720	hypothetical protein, conserved	3 (2.92)		-1,94		0,0969		3,62		0		C	
LinJ.29.1600	hypothetical protein, conserved	3 (5.06)		-2,69		0,0004		1,28		14		M	
LinJ.30.3190	hypothetical protein, conserved	6 (15.57)		1,26		0,0554		1,27		1		M	
LinJ.34.3960	hypothetical protein, conserved	4 (3.68)		1,24		0,0616		1,26		1		M	
LinJ.35.0140	hypothetical protein, conserved	3 (17.65)		1,51		0,0758		1,68		1		M	
LinJ.35.1010	hypothetical protein, conserved	2 (3.71)		-2,11		0,0992		2,98		0		C	
LinJ.36.5310	hypothetical protein, conserved	1 (1.44)		-1,37		0,0491		1,36		0		C	

*^a^*TMDs, transmembrane domains; M, membrane-enriched fraction; C, cytosolic fraction. **^*b*^**Peptide identifications were accepted if they reached greater then 95% probability as specified by the Peptide Prophet algorithm. **^*c*^**TMDs were predicted using TMHMM v2.0.

**Figure 1:**
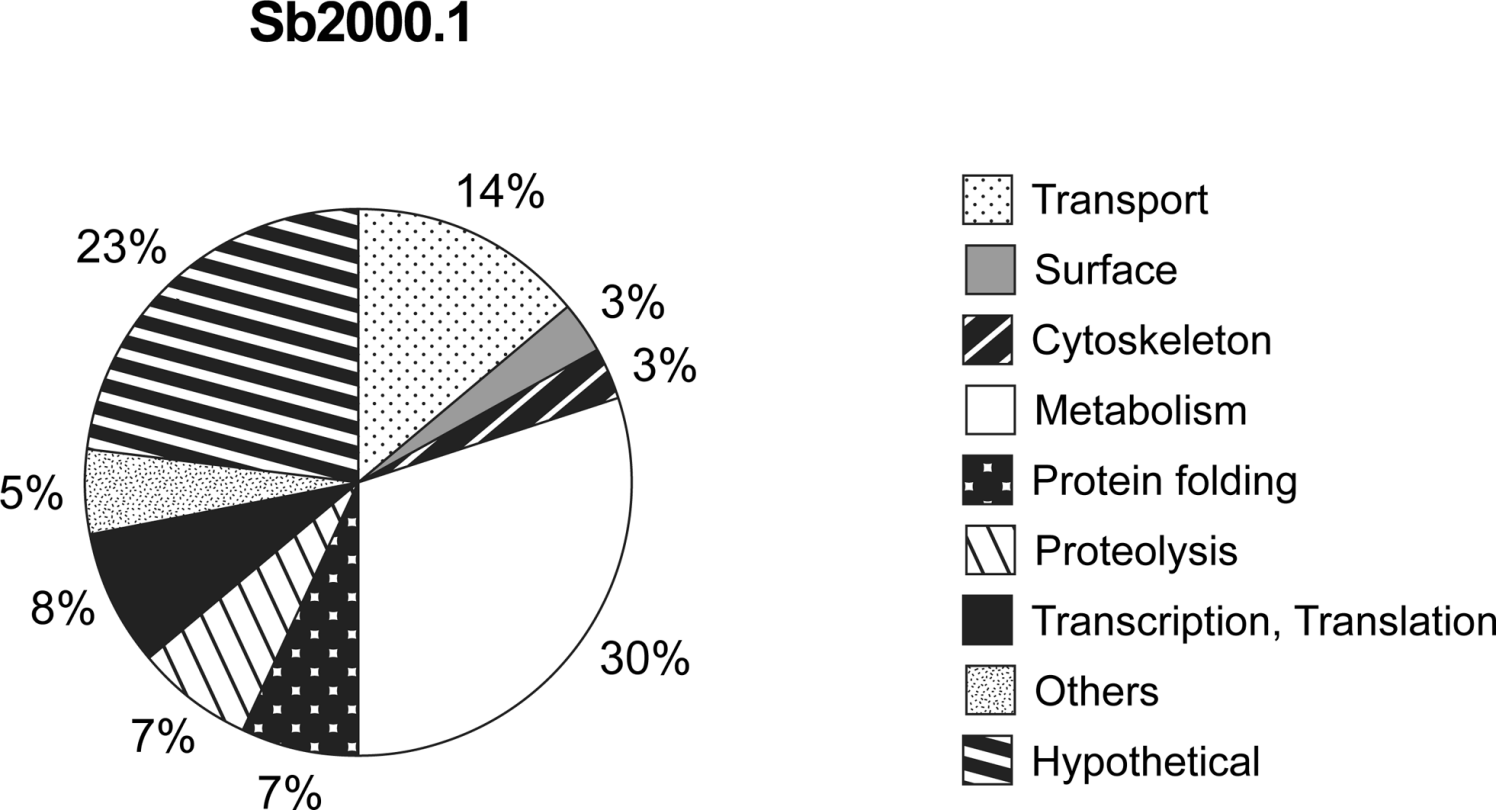
Functional assignment of proteins found differentially expressed by SILAC between *L. infantum* WT and Sb2000.1 mutant. Protein functional classification was based on GeneDB annotations and Gene Ontology.

Differentially expressed proteins with statistical significance were sorted into functional classes according to GeneDB annotations and Gene Ontology. Among the proteins harbouring domains enabling functional assignment, about one third (30%) of the differentially expressed proteins were functionally ascribed to the metabolism group (Figure 1). Hypothetical proteins represented approximately a quarter (23%) of the identified proteins. The third group in importance regrouped proteins involved in transport (14%). The other functional classes (less than 10% each) represented by the proteins identified were transcription/translation, protein folding, proteolysis, cytoskeleton, surface and others (Figure 1). 

Among the metabolic pathways present in our dataset, glycolysis and TCA cycle seemed to be increased in the Sb2000.1 mutant compared to the WT cells (Table 1). Indeed, the glycolytic enzymes hexokinase (LinJ.21.0310), glyceraldehyde 3-phosphate dehydrogenase (LinJ.30.3000) and 2,3-bisphosphoglycerate-independent phosphoglycerate mutase (LinJ.36.6960) were found to be up-regulated in Sb2000.1, whereas the TCA cycle up-regulated enzymes included the NADP-dependant isocitrate dehydrogenase (LinJ.10.0310), the 2-oxoglutarate dehydrogenase E1 component (LinJ.36.3630), GCVL-2 dihydrolipoamide dehydrogenase (LinJ.32.3510), succinate dehydrogenase flavoprotein (LinJ.24.1700) and the fumarate hydratase (LinJ.24.0310). 

The genes coding for candidate proteins being detected either as up- or down-regulated in our proteomic study were cloned into an expression vector and electroporated in *L. infantum* WT and Sb2000.1 cells, respectively (Table S1). The genes of candidate proteins up-regulated in Sb2000.1 were also transfected in a partial revertant strain (Sb2000.1REV) [20] (Table S1). Transfection of the ABC transporter *MRPA* gene (LinJ.23.0290), whose gene product was highlighted for the first time here by a proteomic approach, conferred antimony resistance in WT and partial revertant (Table S1). However, transfection of the 14 other candidate genes failed to show a direct link with SbIII resistance (Table S1). 

### Aneuploidy and amplicon formation lead to drug resistance

Comparative whole genome sequencing allowed the detection of genomic loci amplification and chromosomal aneuploidies in the Sb2000.1 mutant (Figure S1). Indeed, when compared to the WT strain, aneuploidy was observed for 8 chromosomes in Sb2000.1 (chromosomes 9, 11, 12, 20, 21, 23, 26 and 31). It is interesting to note that the copy number of chromosomes 9 and 31 in the mutant appear to be decreased compared to the WT cells, but not constantly across the whole chromosome and not at a level that would reflect the complete deletion of one chromosomal copy in the parasite population (Figure S1). This phenomenon suggests a mosaic aneuploidy (reviewed in [53]) for chromosome 9 in the mutant populations where a proportion of the cells have the same number of chromosomes than the WT and the other proportion have a deleted copy of the chromosome. For chromosome 31 however, approximately two times more reads were obtained in comparison to the other chromosomes in Sb2000.1, which suggests a tetraploid state, and the small decrease in copy number for this chromosome in the mutant could thus result from the loss of one of the four alleles.

Sequencing of Sb2000.1 revealed two amplicons. The first one is derived from chromosome 23 (Figure S1) and encodes the ABC transporter MRPA, hence corroborating our proteomic data where MRPA was shown to be 3.58 times more abundant in Sb2000.1 than in WT cells (Table 1). The second identified amplicon originated from chromosome 17 and contained 12 genes mostly coding for hypothetical proteins (Figure S1). However, we could not link the genes present on this amplicon with either our proteomic results or known resistance genes already described in the literature. Interestingly, we also observed a deleted region at the end of chromosome 6 in Sb2000.1 (Figure S1). In our WT strain, an amplicon originated from this region is naturally present (Ubeda et al., manuscript submitted) and thus the sequencing data support the notion that this amplicon is lost in the antimony mutant (Figure S1). This correlates with our proteomic results where the protein coded by LinJ.06.1340 (Table 1), a gene present on that particular locus of chromosome 6, is 2.95 times less abundant in the Sb2000.1 mutant compared to the WT. Another striking observation made from sequencing data is the apparent deletion in the mutant cells of a region on chromosome 26, which has also been observed in other analysed *Leishmania* resistant mutants (Figure S1). This region contains no gene that we could relate to a general drug resistance mechanism and, since it was observed in many contexts, it may represent a dispensable highly rearranged locus.

### Single nucleotide polymorphisms implicated in antimony resistance

**Figure 2:**
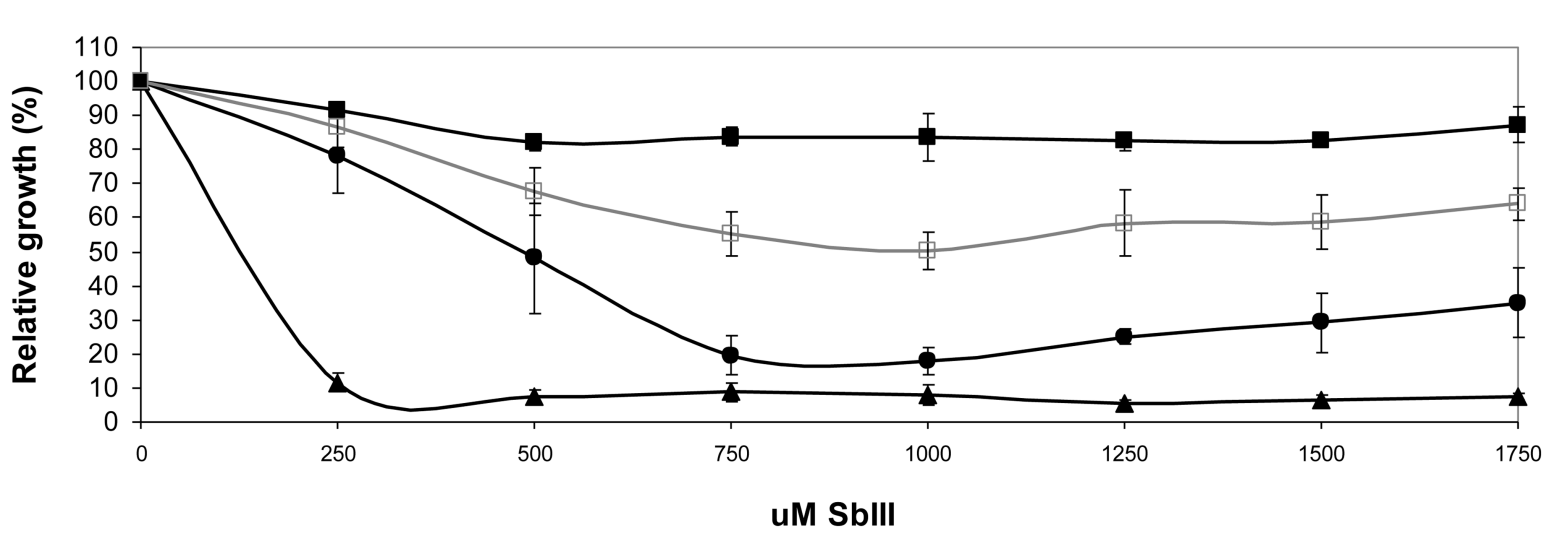
Antimony susceptibilities in Sb2000.1 and Sb2000.1REV transfected with LinJ.33.1810-WT. Growth curves in the presence of increasing drug concentrations were performed by monitoring 72-hour cultures by OD measurement at 600 nm. The average of at least three independent experiments is shown. *L.infantum* WT (filled triangles); Sb2000.1 transfected with pSP72-αNEOα (filled squares); Sb2000.1 transfected with pSP72-αNEOα-LinJ.33.1810WT (open squares); Sb2000.1REV (filled circles).

The *L. infantum* Sb2000.1 mutant remains considerably more resistant to SbIII than its wild-type parent when cultured in the absence of drug pressure for several passages leading to the loss of MRPA amplicons (*L. infantum* Sb2000.1REV in Figure 2) which suggested a role for a stable mutation in resistance. Only few single nucleotide polymorphisms (SNPs) were identified by whole genome sequencing of the antimony resistant mutant, corroborating data obtained on *L. major* [37]. Furthermore, most of the SNPs tested by PCR revealed the presence of polymorphic alleles in the WT, one of them already carrying the resistant mutant allele. These SNPs were not further analysed. However, one homozygous SNP in a gene coding for a protein kinase (LinJ.33.1810) was confirmed by conventional PCR and DNA resequencing. This mutation consists of a guanine to adenine transition at gene position 1885 giving rise to a change from a glutamic acid to a lysine at position 629 in the protein. It is interesting to note that the glutamic acid at this position is conserved in all other sequenced *Leishmania* species. To assess the role of this SNP in the resistance phenotype, we cloned the WT version of LinJ.33.1810 and transfected it in either *L. infantum* WT or Sb2000.1 cells. No phenotype was observed when the gene was overexpressed in WT cells (data not shown), but transfection of the WT copy of this protein kinase coding gene sensitized moderately but significantly the Sb2000.1 mutant to SbIII (Figure 2), hence confirming the link with the antimony resistance phenotype.

## Discussion

Pentavalent antimonials (SbV) have been the first-line drugs for the treatment of leishmaniasis for almost the last seven decades but clinical resistance to these drugs has emerged as an obstacle to successful treatment, especially in India [3-5]. Using whole genome sequencing and SILAC methodologies, we probed the genomic and proteomic changes induced by SbIII in an *in vitro* generated *L. infantum* resistant mutant. Since membrane proteins are often underrepresented in proteomic studies, we chose to analyse SILAC-labeled ME and cytosolic proteins separately in order to increase the proteome coverage. Many of the identified proteins were hypothetical and thus will need further investigation of their potential role in drug resistance. Proteomic analysis of membrane proteins has allowed the detection of MRPA, a known protein in SbIII resistance phenotype [16-22]. As previously shown [20], transfection of this gene in WT cells gave rise to an intermediary SbIII resistance phenotype (Table S1). However, transfection of 14 other candidate genes coding for proteins differentially expressed failed to show a direct involvement in drug resistance (Table S1). This suggests that the proteins identified here are either involved in drug resistance by an indirect mean or that a combination of some of these proteins is required to obtain a resistance phenotype. It is also possible that some of the differences between these isogenic parasites are not related to antimony resistance and were due to the mutagenic nature of the drug. One possible approach to decrease the false-negative discovery rate would be to study more than one mutant and concentrate on recurrent mutations in independent mutants.

The surface metallopeptidase GP63 had a net decrease in its expression in the Sb2000.1 mutant (Table 1). This protein has already been shown to be down-regulated in tunicamycin-resistant *L. chagasi* [54], but up-regulated in sinefungin-resistant *L. donovani* and *L. tropica* [55]. Similarly, the highly immunogenic amastin surface glycoproteins were previously reported as being modified at the parasite cell surface in several clinical *Leishmania* strains isolated from unresponsive patients to antimonials [36]. Our SILAC data indicated a decrease in the protein levels of one amastin (LinJ.30.0920) (Table 1). Histone H4 (LinJ.36.0020), that was more abundant in Sb2000.1 at the protein level, has previously been shown to be overexpressed at the RNA level in antimony-resistant *L. donovani* field isolates [56].

The levels of heat-shock proteins and chaperonins are also known to be increased in the presence of antimony and its related metal arsenite [57]. The heat-shock protein hsp70, which is already known to increase antimony tolerance [58], was indeed up-regulated in the resistant mutant as well as the HSP60 chaperonin (Table 1). A number of other heat-shock proteins and chaperonins were also detected as overproduced in *L. infantum* Sb2000.1 (Table 1). While not experimentally tested, these proteins are more likely to be implicated in a more general drug/stress response mechanism.

Key energy metabolism pathways were up-regulated in the Sb2000.1 mutant (Table 1). The observed modulation of glycolytic enzymes is supported by a previous report [34] and may correspond to a general stress response mechanism. TCA cycle is also up-regulated in the SbIII resistant mutant (Table 1). 

Some evidences have shown that antimony kills the parasite by a caspase-independent apoptosis-like process involving DNA fragmentation [59-61]. In line with these observations, an interesting protein down-regulated in our SbIII mutant was the calpain-like cysteine peptidase SKCRP14.1 (LinJ.14.0920). This protein has already been shown to be down-regulated in a *L. donovani* field strain resistant to SbV and has been proposed to be a regulator of drug-mediated programmed cell death [31]. 

While aneuploidy is a well-known drug resistance mechanism in cancer cells (reviewed in [62]) and pathogenic yeasts [63], as well as in *Leishmania* [20,64], we could not link our observed CNVs by whole genome sequencing with our proteomic results. This is nonetheless consistent with a previous study in yeast where it was shown that an extra copy of a chromosome did not necessarily lead to an increase in proteins encoded by the genes present on this specific chromosome [65]. In yeast, either the extra mRNA molecules are not translated or the protein products are degraded shortly after synthesis [65]. The same principle may apply for *Leishmania*, thus explaining the non-concordance of CNVs and protein levels detected in Sb2000.1 parasites.

Two amplicons respectively derived from chromosomes 17 and 23 were detected from our sequencing data in the SbIII resistant mutant (Figure S1). The resistance phenotype was partially reversible in Sb2000.1 after only 30 passages in the absence of antimony, mainly due to the loss of the amplicon derived from chromosome 23 coding for the ABC transporter MRPA [20].

Our sequencing approach identified a low level of SNPs in the resistant mutant and only one SNP was confirmed when tested by PCR and conventional DNA sequencing. Transfection of the WT version of the protein kinase coded by LinJ.33.1810 in Sb2000.1 conferred a modest but reproducible and significant sensitization of the mutant (Figure 2). Antimony is a known protein phosphatase inhibitor [66], and a mutation in a protein kinase may compensate for the inhibition of a phosphatase. Also, downregulation of protein kinase has recently been linked [67] and associated [68] to resistance to antimony in *Leishmania* field isolates and it is possible that the mutation reported here serves similar purposes. While mutations are usually stable, the LinJ.33.1810-E629K allele was not observed in Sb2000.1REV possibly by the selection of a minor subpopulation within the mutant whole population. This mutation ‘reversion’, along with the loss of the MRPA amplicon, may explain the decrease in resistance for the revertant line. 

In conclusion, our study has revealed that CNVs, amplicons, point mutations and changes in protein expression are associated with drug resistance. Several proteins were identified as being significantly modulated in the Sb2000.1 mutant and our proteomic study has reinforced the impact of SbIII resistance on several important metabolic pathways in *Leishmania*. This study has also reinforced the role of MRPA, identified for the first time in a proteomic study, as a key player in the antimony resistance. Furthermore, aneuploidy for 8 chromosomes and a SNP in a gene coding for a protein kinase have been correlated to antimony resistance.

## Supporting Information

Figure S1 **Chomosomal copy number variations in Sb2000.1 compared to WT.** Chromosomes were divided into genomic windows of 5kb and the number of reads mapping to each windows determined in *L. infantum* Sb2000.1 and WT. The Sb2000.1/WT log_2_ ratios of read counts were then plotted for each genomic window on a per chromosome basis.
(PDF)

Table S1 **Genes whose expression of the protein product was significantly modulated in *L. infantum* Sb2000.1 compared to *L. infantum* WT in the SILAC experiments were functionally characterized for their role in resistance by gene transfection experiments.**
(XLS)
