# Supplementary material for: Proteomic and Genomic Analyses of Antimony Resistant Leishmania infantum Mutant
Source: PLoS One. 2013 Nov 27;8(11):e81899. doi: 10.1371/journal.pone.0081899 (PMC3842243; doi:10.1371/journal.pone.0081899)
Supplement: Figure S1 — Chomosomal copy number variations in Sb2000.1 compared to WT. Chromosomes were divided into genomic windows of 5kb and the number of reads mapping to each windows determined in L. infantum Sb2000.1 and WT. The Sb2000.1/WT log2 ratios of read counts were then plotted for each genomic window on a per chromosome basis. (PDF) [file pone.0081899.s001.pdf]

Figure S1 : Chomosomal copy number variations in Sb2000.1 compared to WT

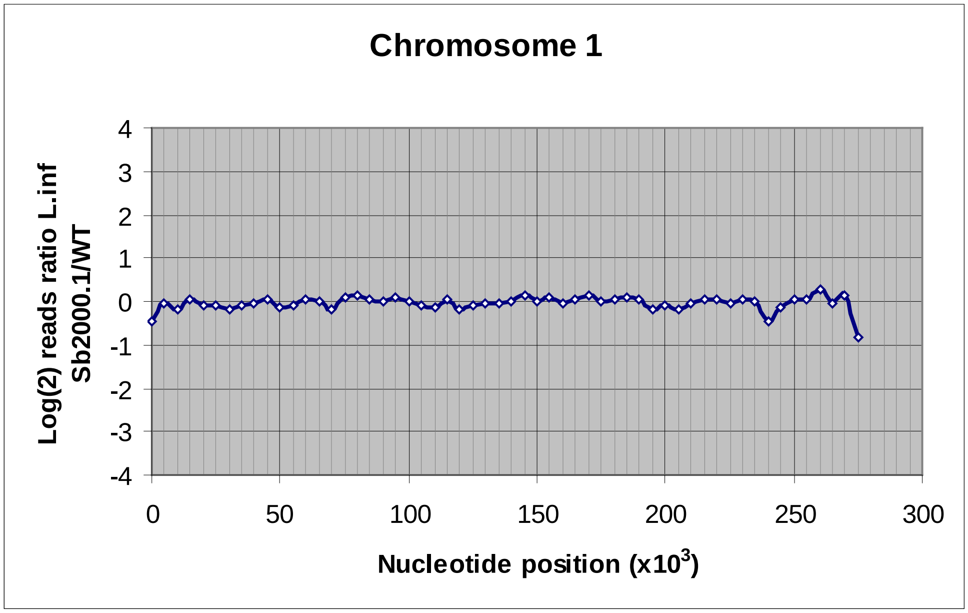

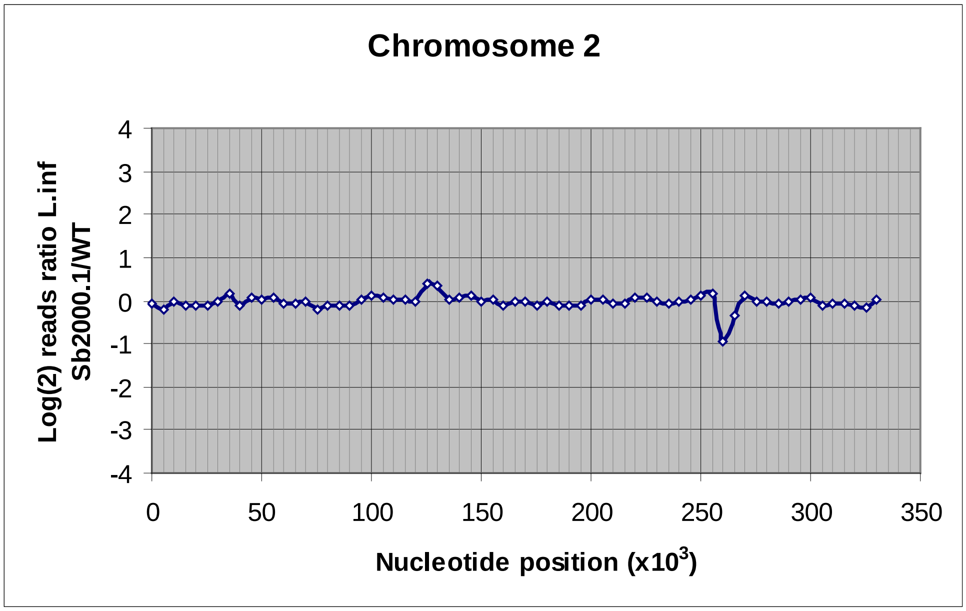

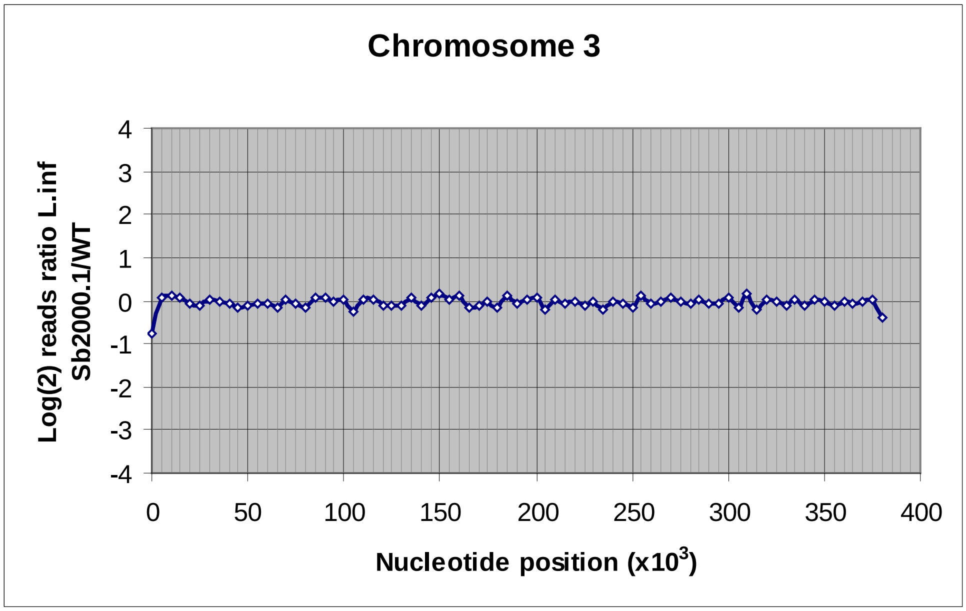
Chromosome 3
Log(2) reads ratio L.inf Sb2000.1/WT
4
3
2
1
0
-1
-2
-3
-4
0
50
100
150
200
250
300
350
400
Nucleotide position (x10^3)

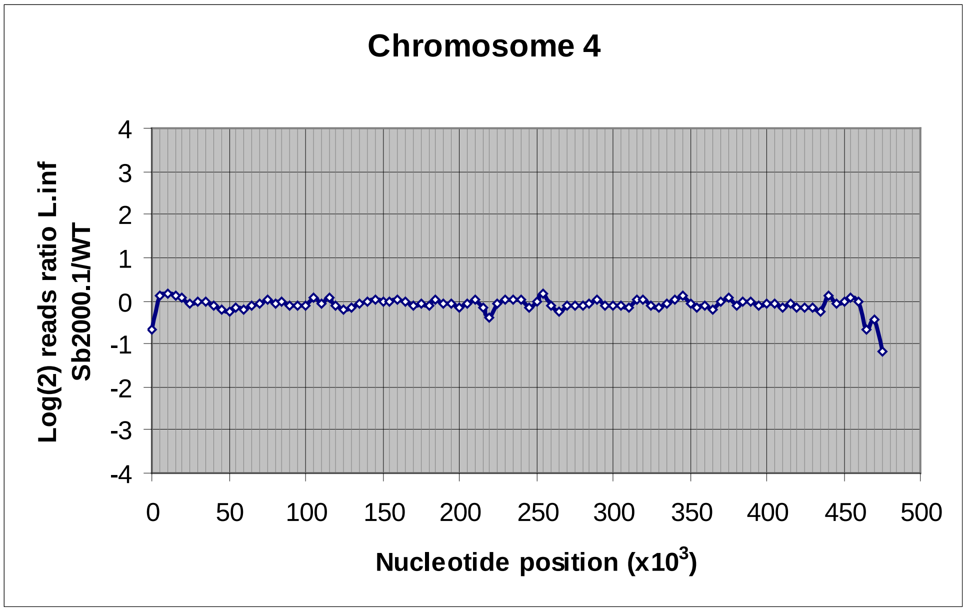
Chromosome 4
Log(2) reads ratio L.inf Sb2000.1/WT
4
3
2
1
0
-1
-2
-3
-4
0
50
100
150
200
250
300
350
400
450
500
Nucleotide position (x10^3)

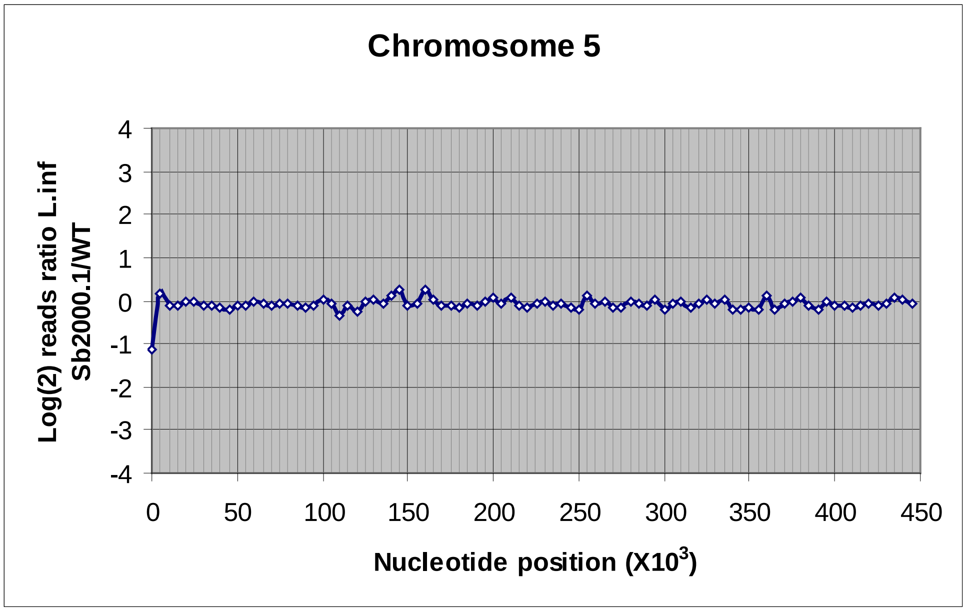
Chromosome 5
Log(2) reads ratio L.inf Sb2000.1/WT
4
3
2
1
0
-1
-2
-3
-4
0
50
100
150
200
250
300
350
400
450
Nucleotide position (X10^3)

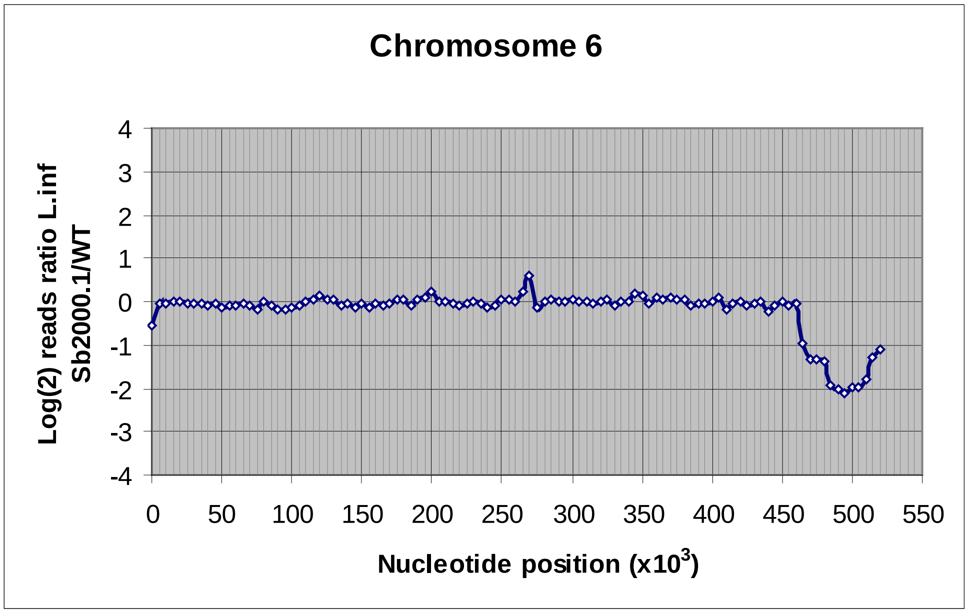
Chromosome 6
Log(2) reads ratio L.inf Sb2000.1/WT
4
3
2
1
0
-1
-2
-3
-4
0
50
100
150
200
250
300
350
400
450
500
550
Nucleotide position (x10^3)

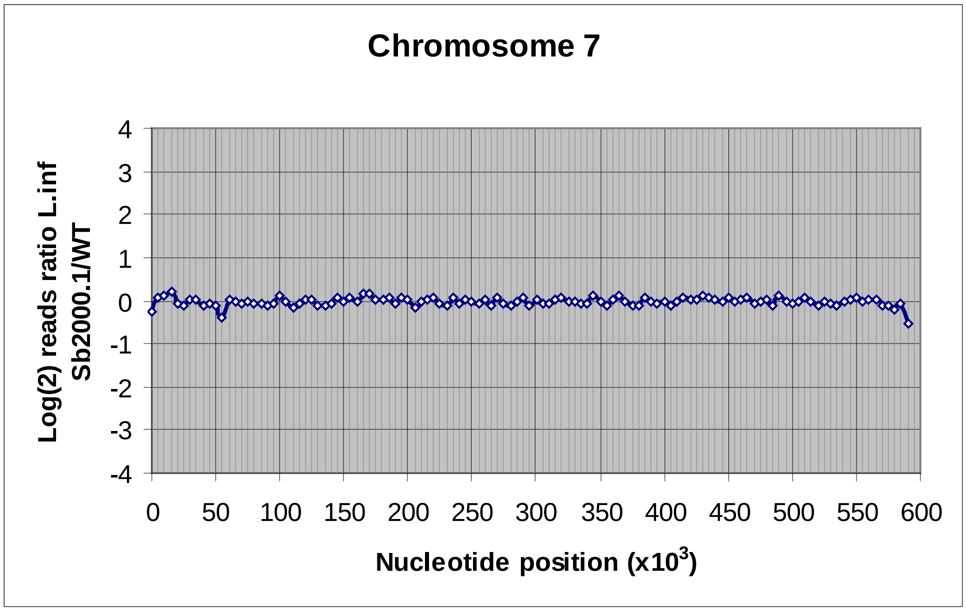
Chromosome 7
Log(2) reads ratio L.inf Sb2000.1/WT
4
3
2
1
0
-1
-2
-3
-4
0
50
100
150
200
250
300
350
400
450
500
550
600
Nucleotide position (x10³)

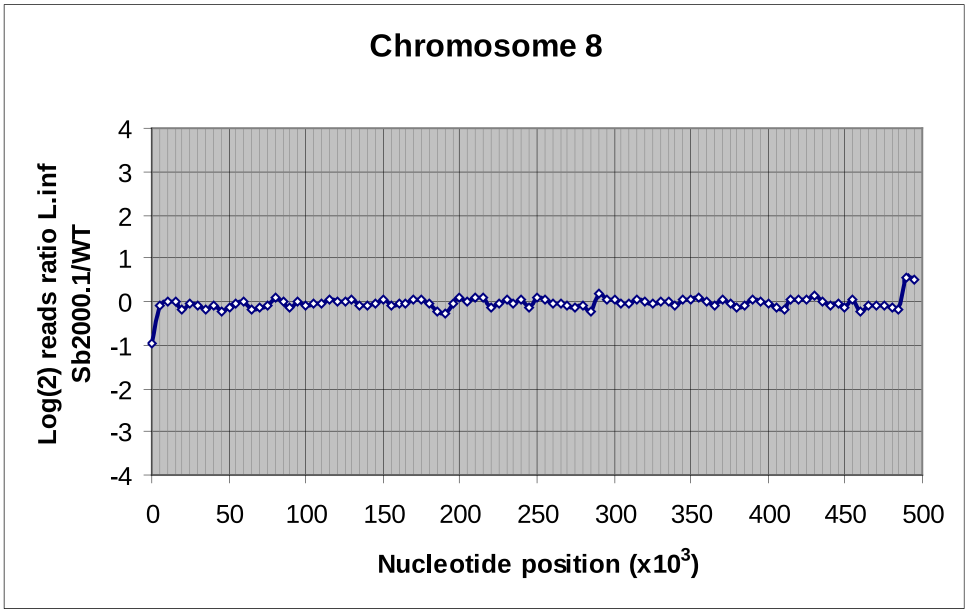
Chromosome 8
Log(2) reads ratio L.inf Sb2000.1/WT
4
3
2
1
0
-1
-2
-3
-4
0
50
100
150
200
250
300
350
400
450
500
Nucleotide position (x10³)

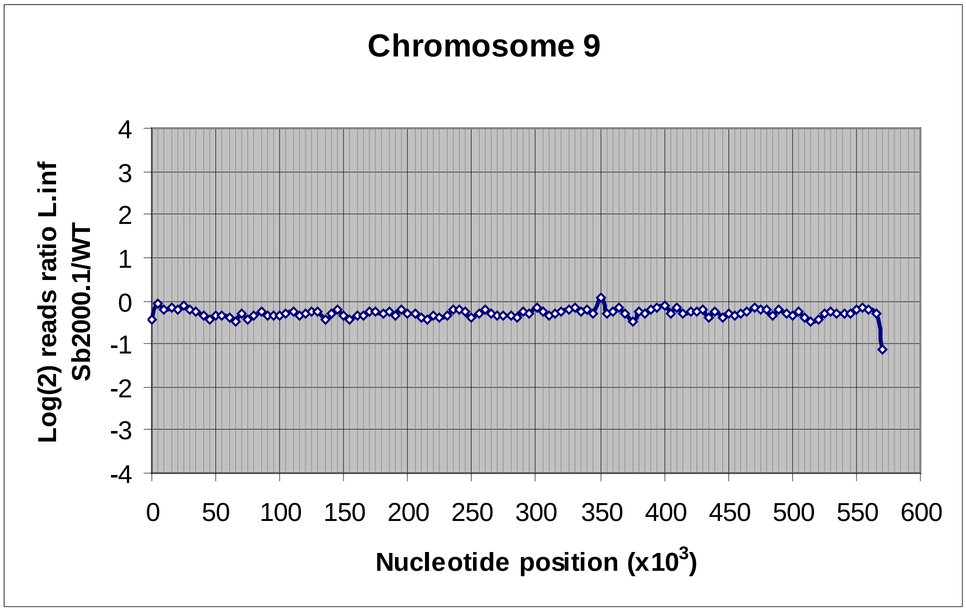
Chromosome 9
Log(2) reads ratio L.inf Sb2000.1/WT
4
3
2
1
0
-1
-2
-3
-4
0
50
100
150
200
250
300
350
400
450
500
550
600
Nucleotide position (x10³)

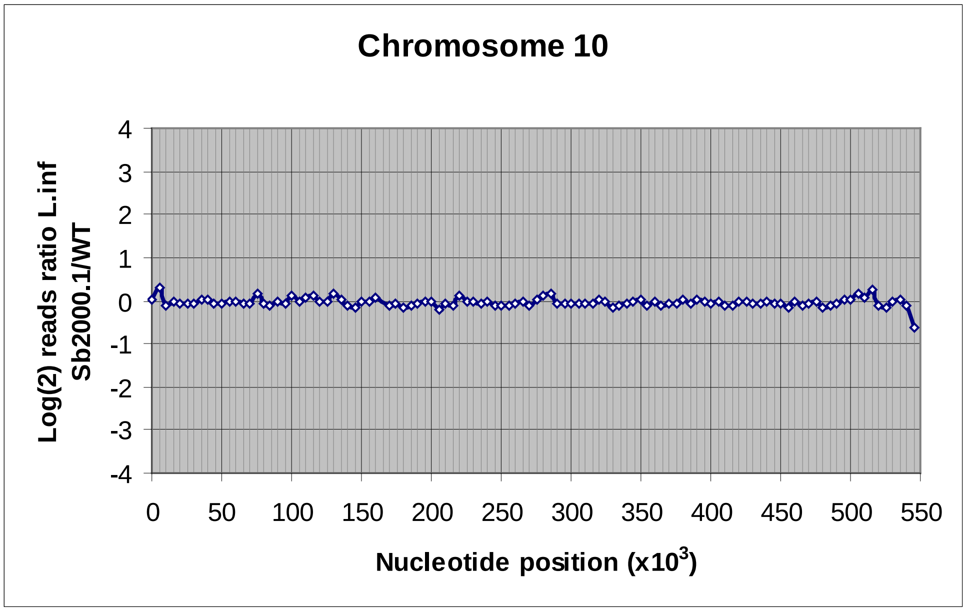
Chromosome 10
Log(2) reads ratio L.inf Sb2000.1/WT
4
3
2
1
0
-1
-2
-3
-4
0
50
100
150
200
250
300
350
400
450
500
550
Nucleotide position (x10³)

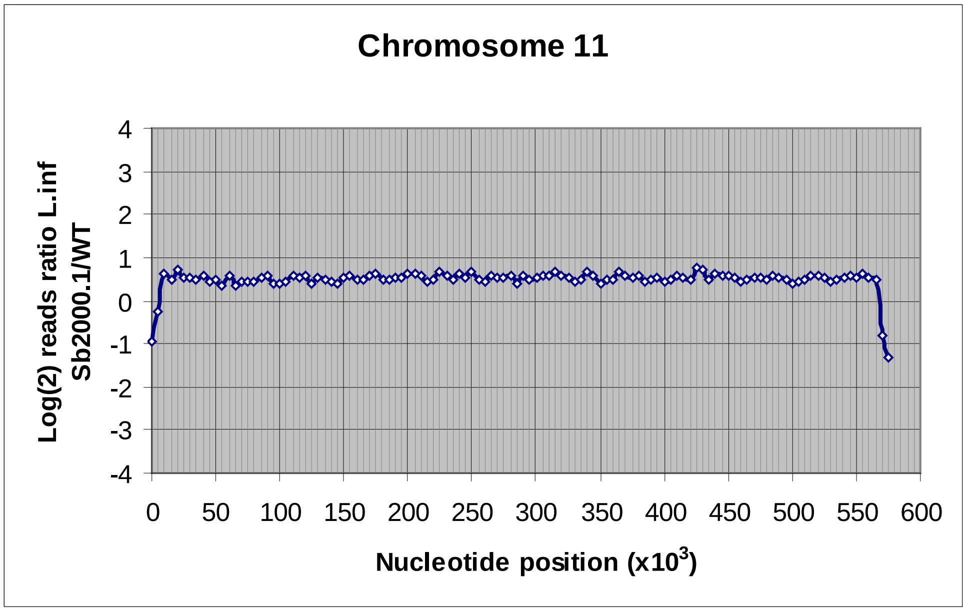
Chromosome 11
Log(2) reads ratio L.inf Sb2000.1/WT
4
3
2
1
0
-1
-2
-3
-4
0
50
100
150
200
250
300
350
400
450
500
550
600
Nucleotide position (x10³)

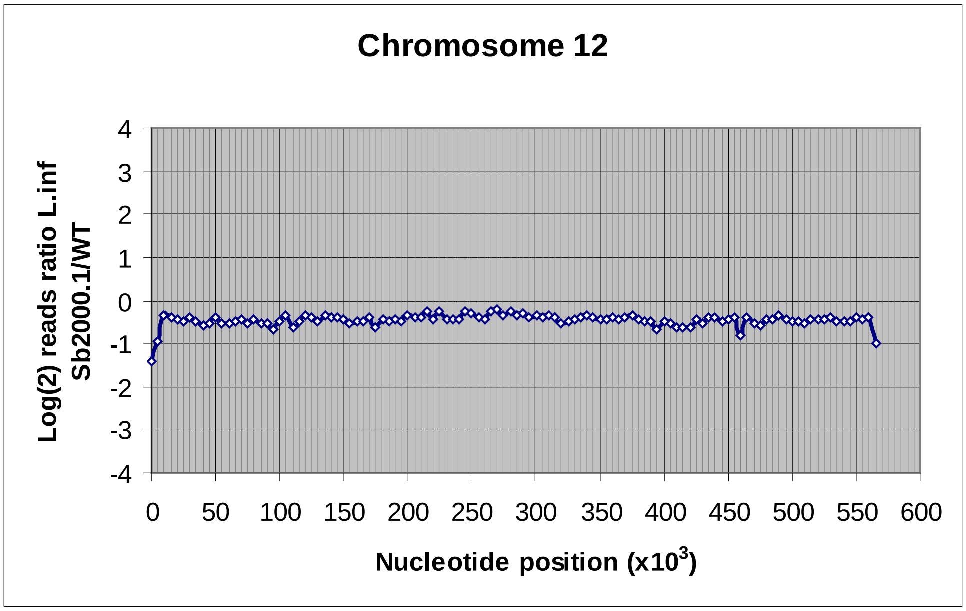
Chromosome 12
Log(2) reads ratio L.inf Sb2000.1/WT
4
3
2
1
0
-1
-2
-3
-4
0
50
100
150
200
250
300
350
400
450
500
550
600
Nucleotide position (x10³)

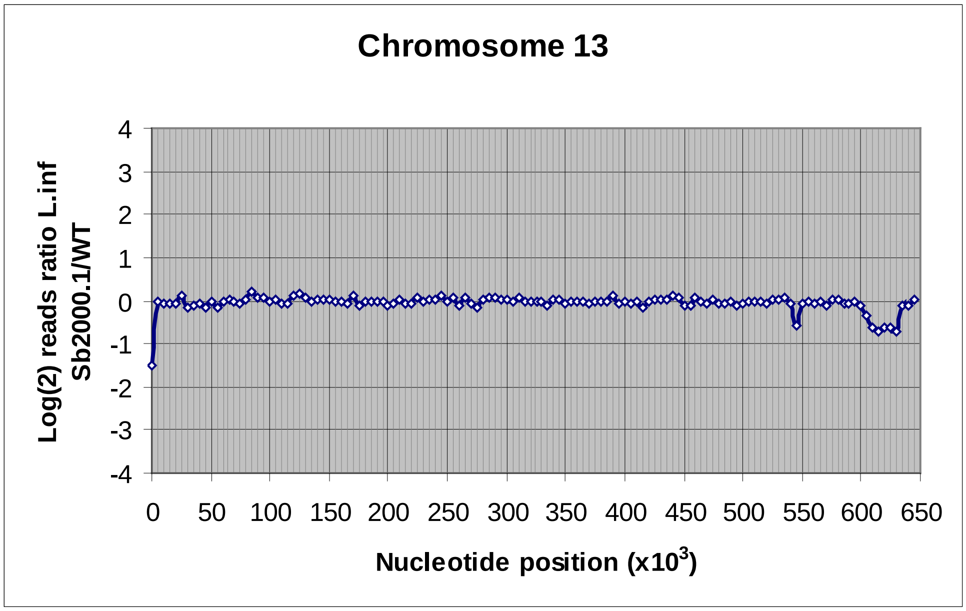
Chromosome 13
Log(2) reads ratio L.inf Sb2000.1/WT
4
3
2
1
0
-1
-2
-3
-4
0 50 100 150 200 250 300 350 400 450 500 550 600 650
Nucleotide position (x10^3)

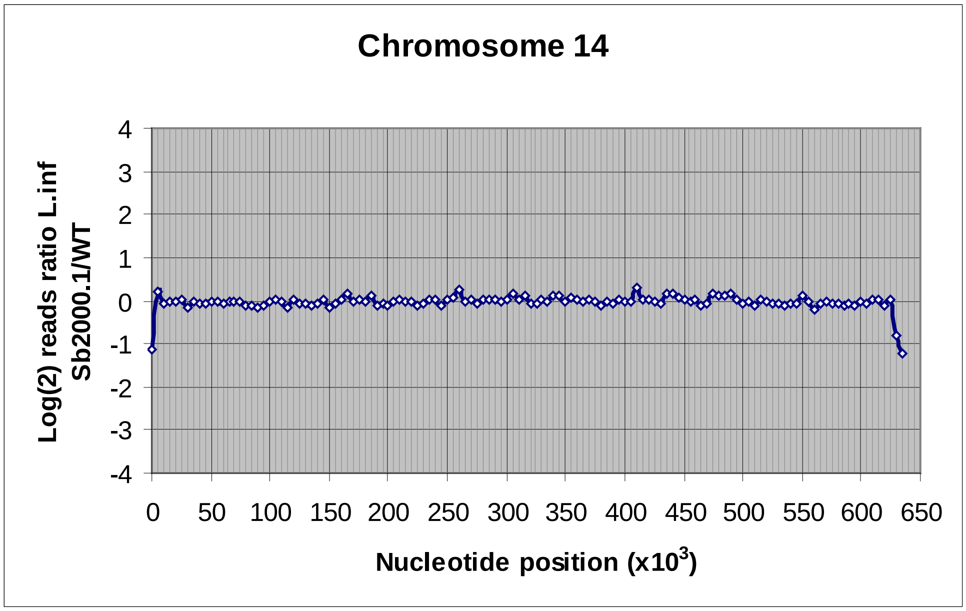
Chromosome 14
Log(2) reads ratio L.inf Sb2000.1/WT
4
3
2
1
0
-1
-2
-3
-4
0 50 100 150 200 250 300 350 400 450 500 550 600 650
Nucleotide position (x10^3)

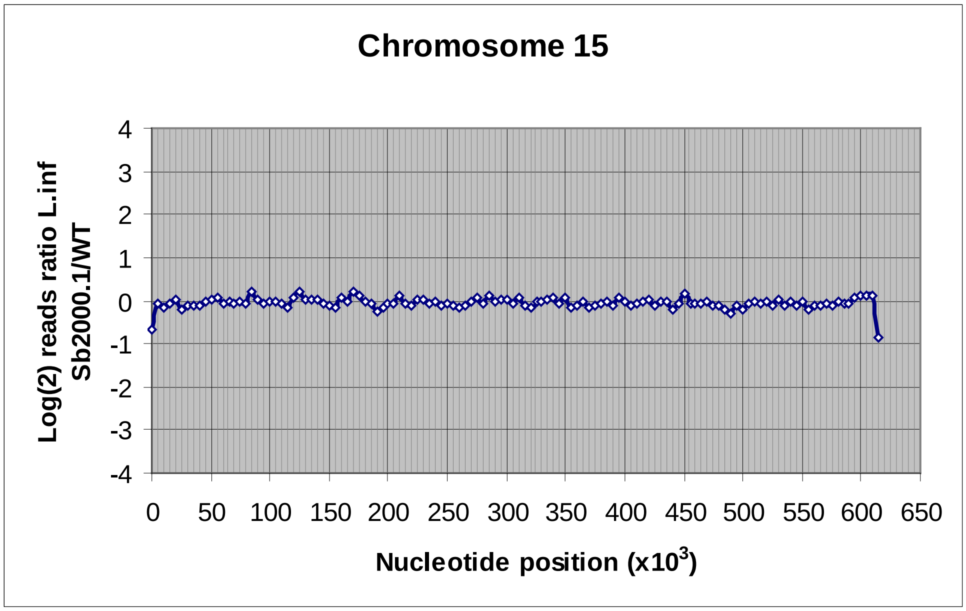
Chromosome 15
Log(2) reads ratio L.inf Sb2000.1/WT
4
3
2
1
0
-1
-2
-3
-4
0 50 100 150 200 250 300 350 400 450 500 550 600 650
Nucleotide position (x10³)

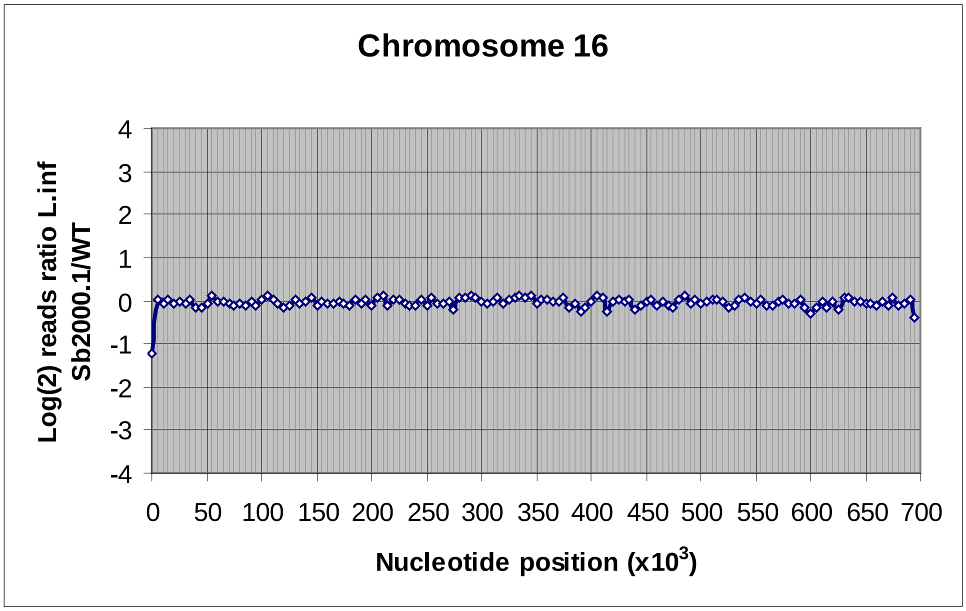
Chromosome 16
Log(2) reads ratio L.inf Sb2000.1/WT
4
3
2
1
0
-1
-2
-3
-4
0 50 100 150 200 250 300 350 400 450 500 550 600 650 700
Nucleotide position (x10³)

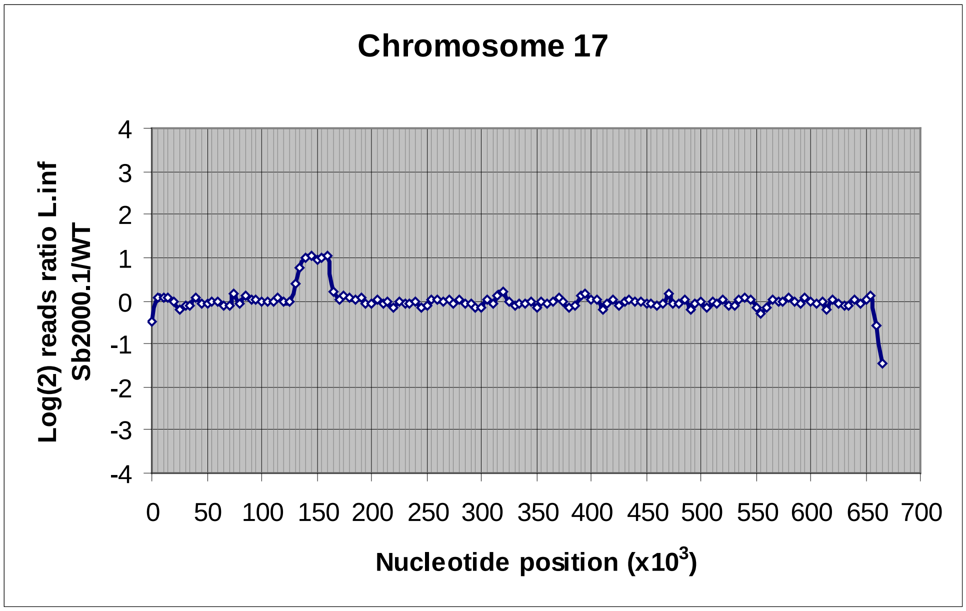

Chromosome 17
Log(2) reads ratio L.inf Sb2000.1/WT
4
3
2
1
0
-1
-2
-3
-4
0 50 100 150 200 250 300 350 400 450 500 550 600 650 700
Nucleotide position (x10³)

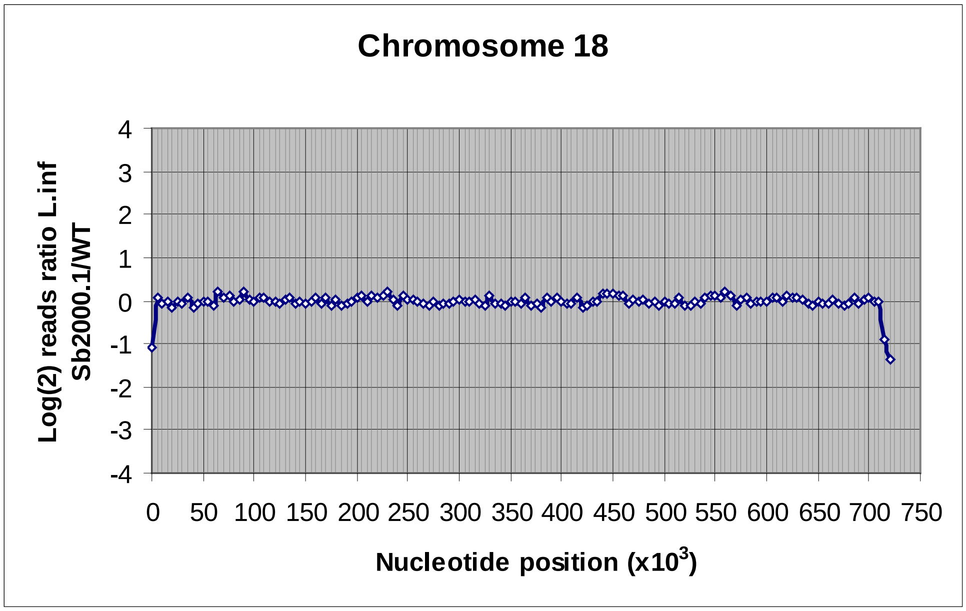

Chromosome 18
Log(2) reads ratio L.inf Sb2000.1/WT
4
3
2
1
0
-1
-2
-3
-4
0 50 100 150 200 250 300 350 400 450 500 550 600 650 700 750
Nucleotide position (x10³)

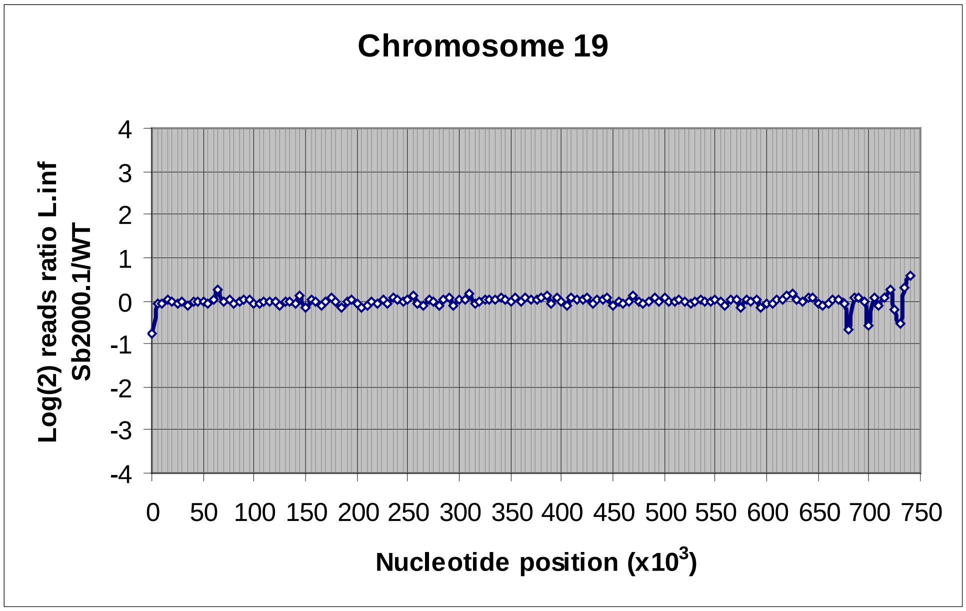
Chromosome 19
Log(2) reads ratio L.inf Sb2000.1/WT
4
3
2
1
0
-1
-2
-3
-4
0 50 100 150 200 250 300 350 400 450 500 550 600 650 700 750
Nucleotide position (x10^3)

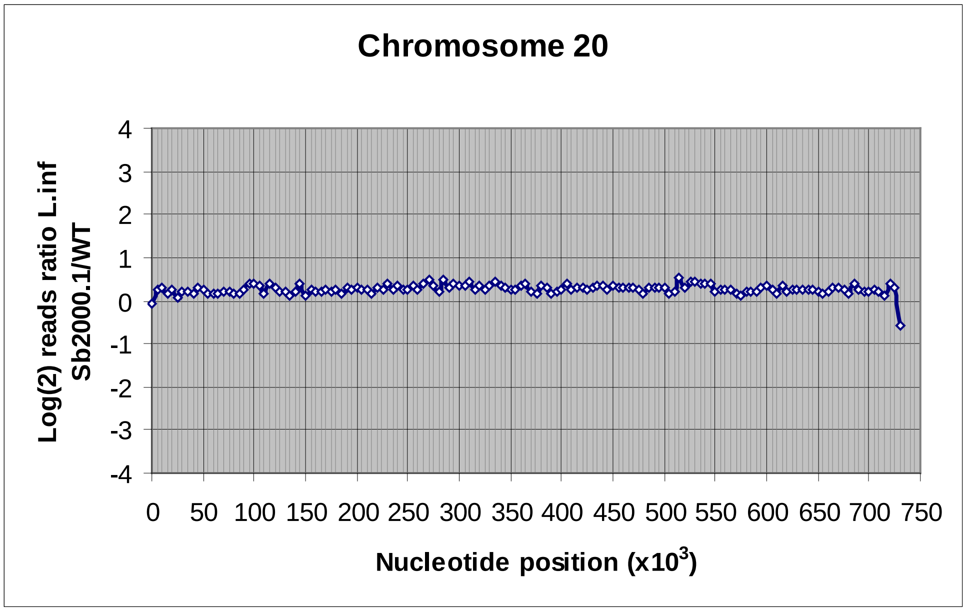
Chromosome 20
Log(2) reads ratio L.inf Sb2000.1/WT
4
3
2
1
0
-1
-2
-3
-4
0 50 100 150 200 250 300 350 400 450 500 550 600 650 700 750
Nucleotide position (x10^3)

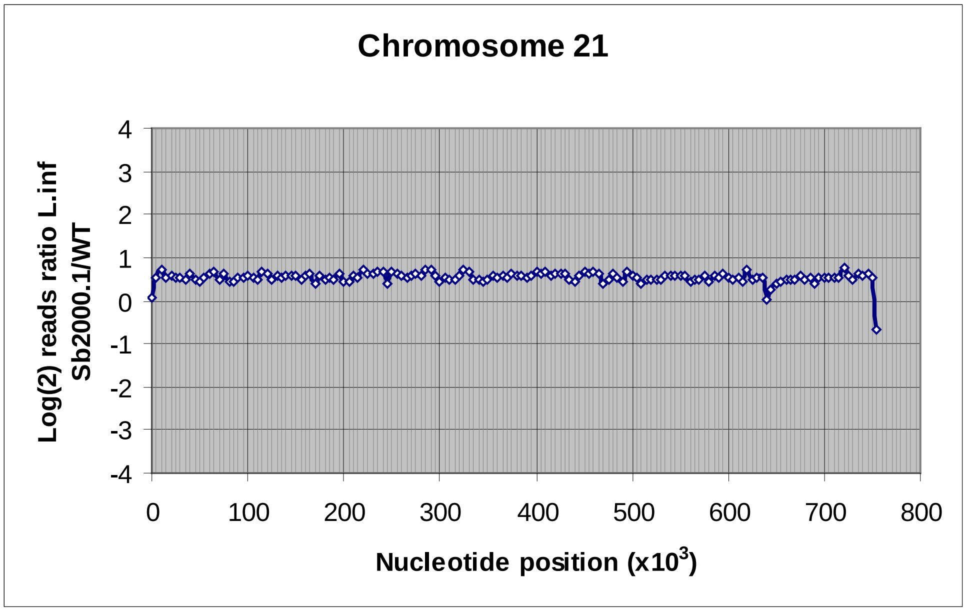
Chromosome 21
Log(2) reads ratio L.inf Sb2000.1/WT
4
3
2
1
0
-1
-2
-3
-4
0
100
200
300
400
500
600
700
800
Nucleotide position (x10^3)

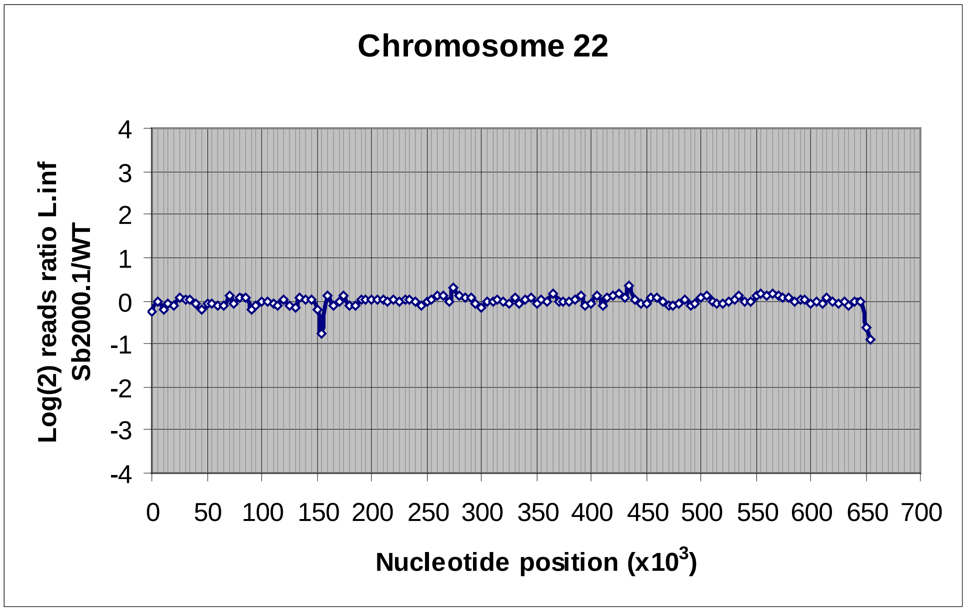
Chromosome 22
Log(2) reads ratio L.inf Sb2000.1/WT
4
3
2
1
0
-1
-2
-3
-4
0
50
100
150
200
250
300
350
400
450
500
550
600
650
700
Nucleotide position (x10^3)

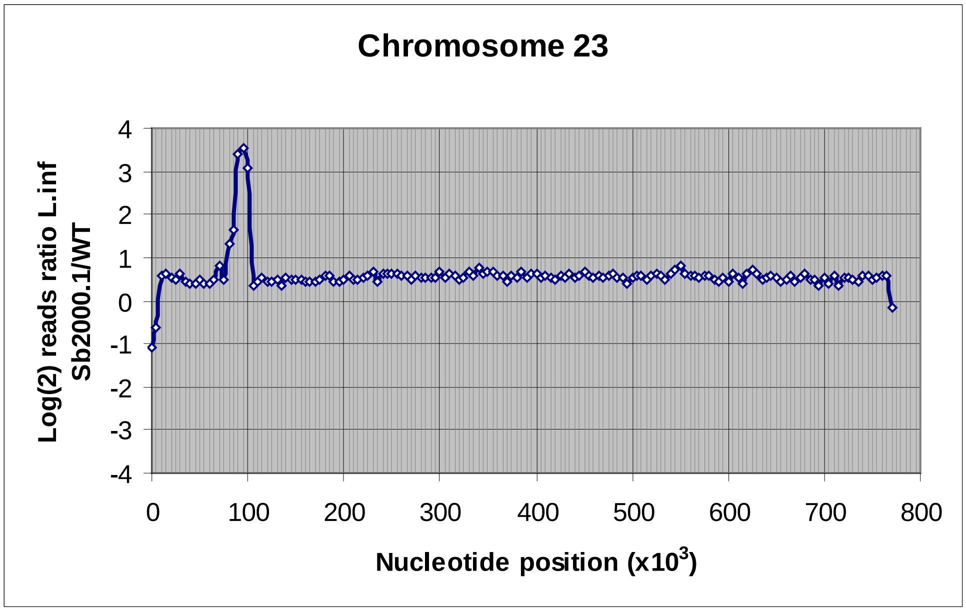
Chromosome 23
Log(2) reads ratio L.inf Sb2000.1/WT
4
3
2
1
0
-1
-2
-3
-4
0
100
200
300
400
500
600
700
800
Nucleotide position (x10^3)

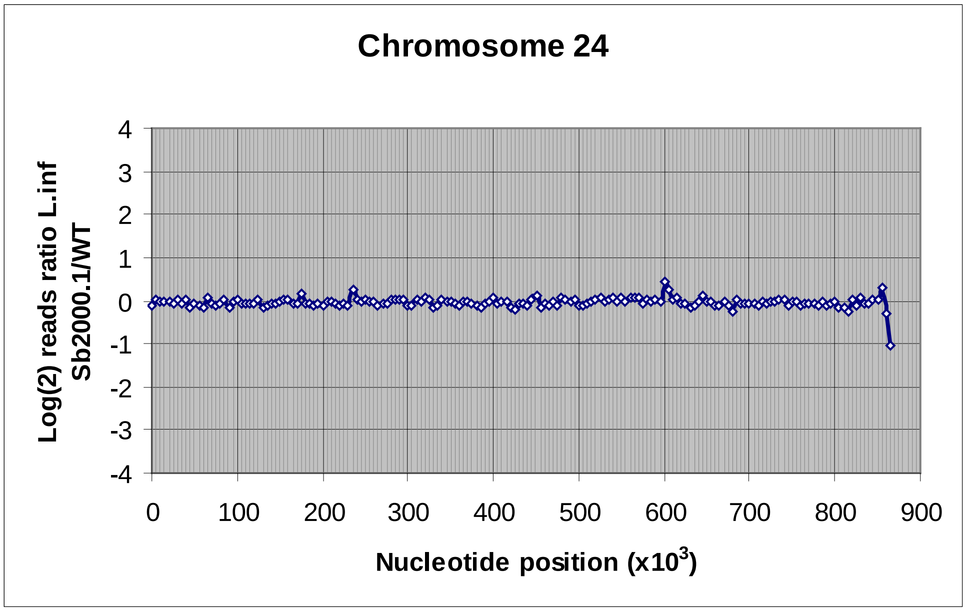
Chromosome 24
Log(2) reads ratio L.inf Sb2000.1/WT
4
3
2
1
0
-1
-2
-3
-4
0
100
200
300
400
500
600
700
800
900
Nucleotide position (x10^3)

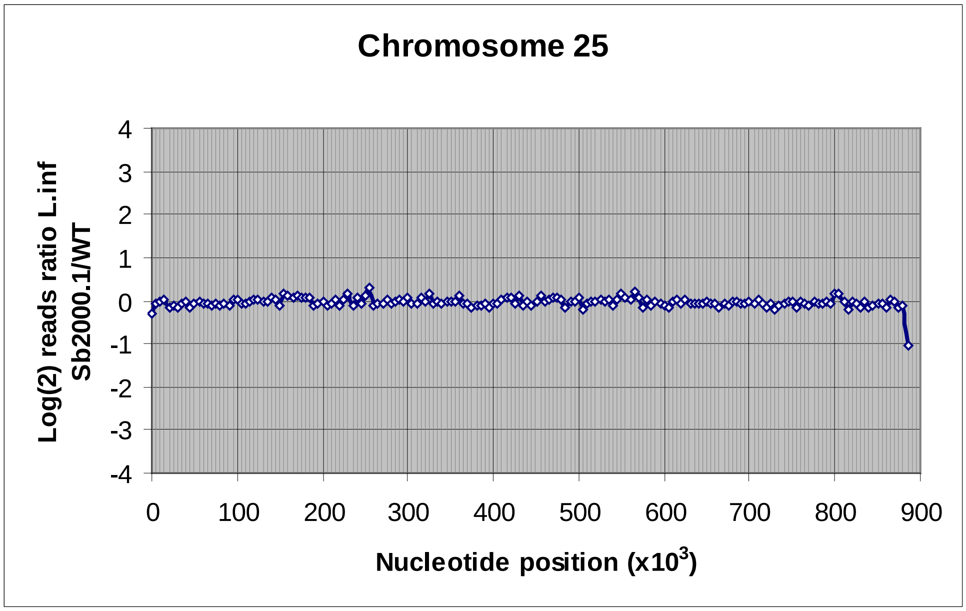
Chromosome 25
Log(2) reads ratio L.inf Sb2000.1/WT
4
3
2
1
0
-1
-2
-3
-4
0
100
200
300
400
500
600
700
800
900
Nucleotide position (x10³)

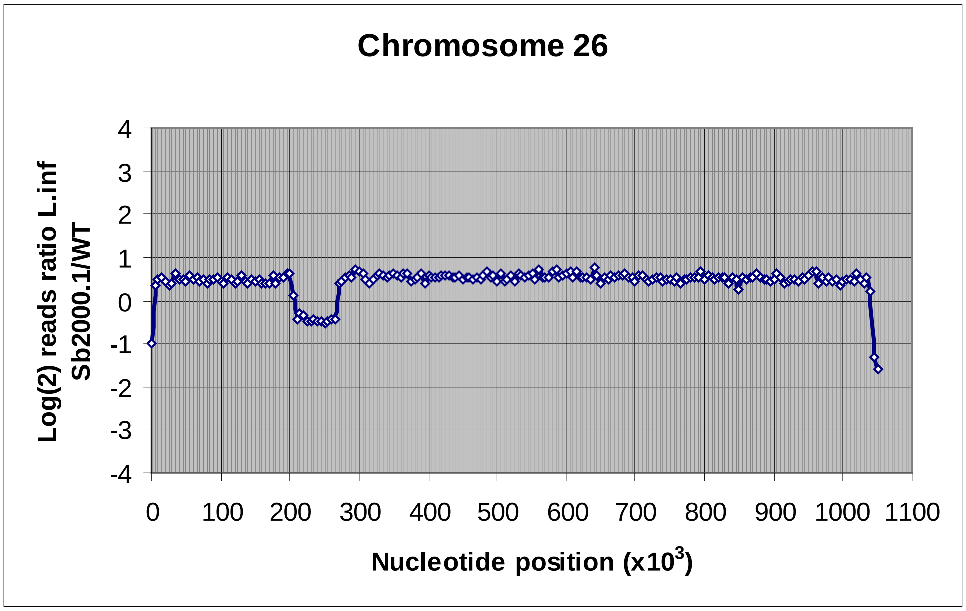
Chromosome 26
Log(2) reads ratio L.inf Sb2000.1/WT
4
3
2
1
0
-1
-2
-3
-4
0
100
200
300
400
500
600
700
800
900
1000
1100
Nucleotide position (x10³)

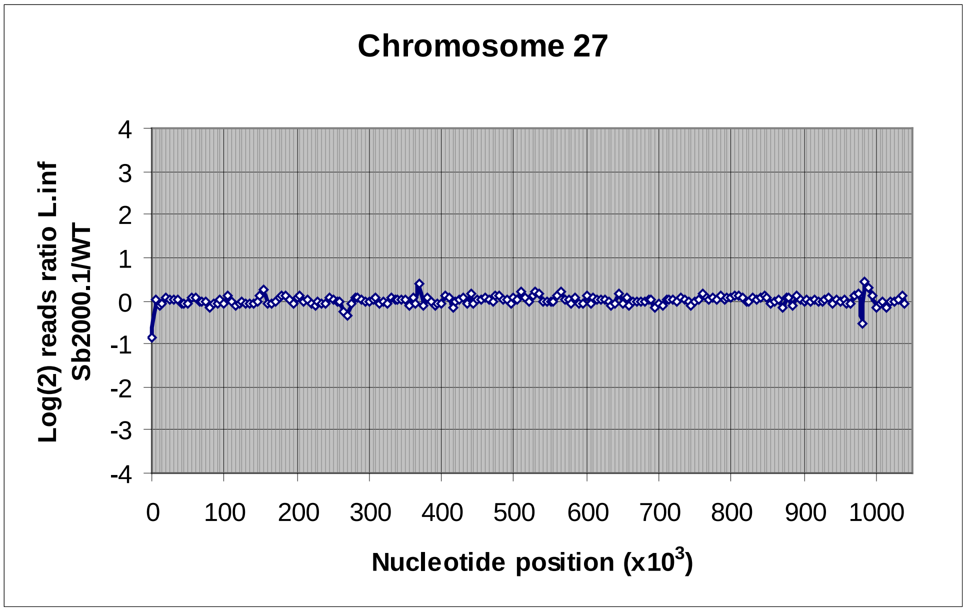
Chromosome 27
4
3
2
1
0
-1
-2
-3
-4
0 100 200 300 400 500 600 700 800 900 1000
Log(2) reads ratio L.inf Sb2000.1/WT
Nucleotide position (x10³)

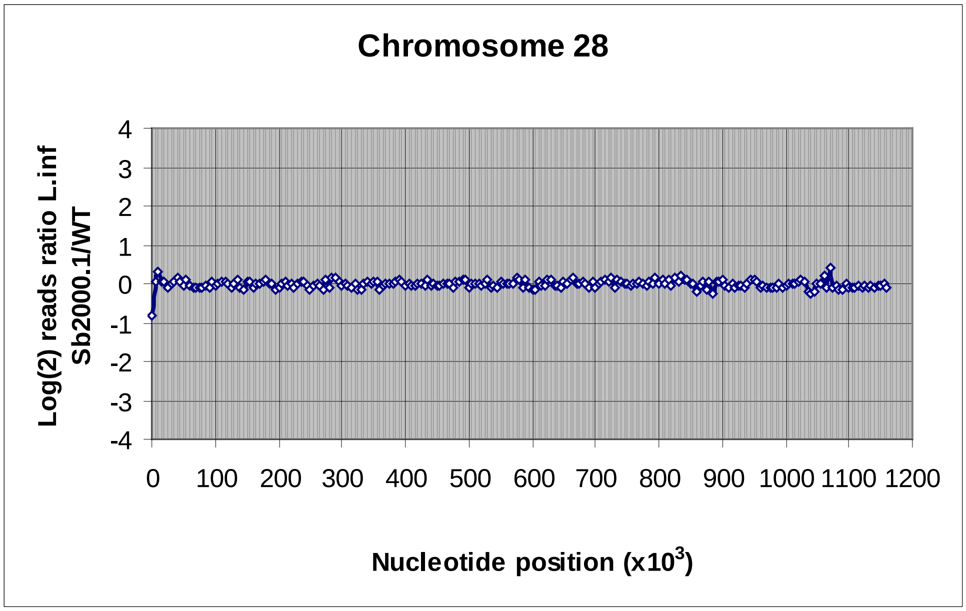
Chromosome 28
4
3
2
1
0
-1
-2
-3
-4
0 100 200 300 400 500 600 700 800 900 1000 1100 1200
Log(2) reads ratio L.inf Sb2000.1/WT
Nucleotide position (x10³)

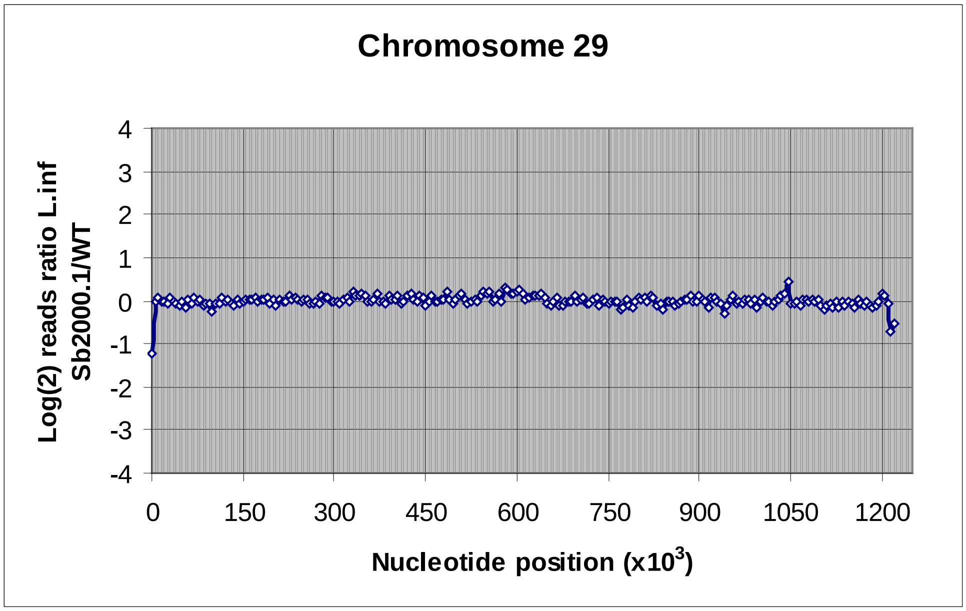
Chromosome 29
Log(2) reads ratio L.inf Sb2000.1/WT
4
3
2
1
0
-1
-2
-3
-4
0
150
300
450
600
750
900
1050
1200
Nucleotide position (x10^3)

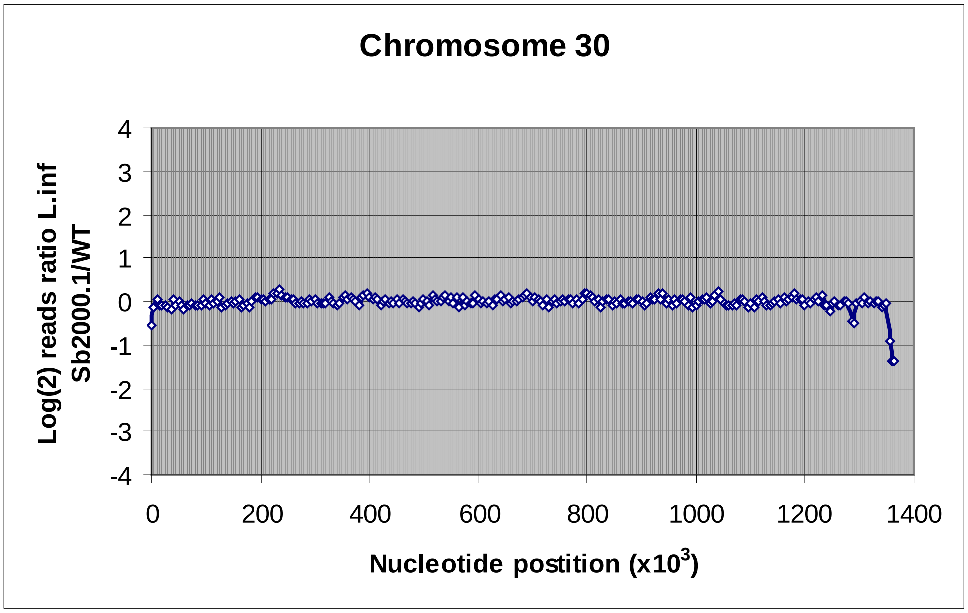
Chromosome 30
Log(2) reads ratio L.inf Sb2000.1/WT
4
3
2
1
0
-1
-2
-3
-4
0
200
400
600
800
1000
1200
1400
Nucleotide postition (x10^3)

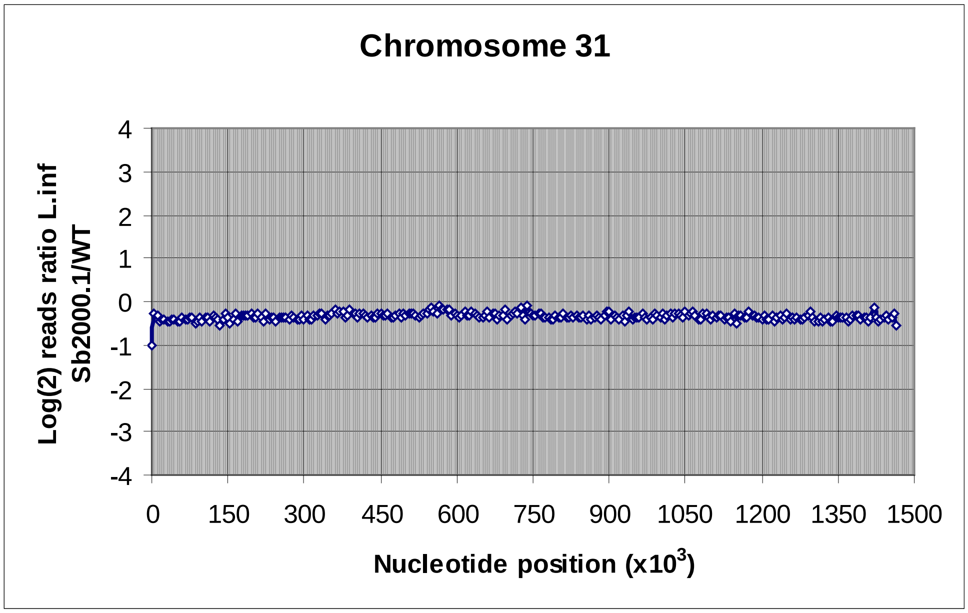
Chromosome 31
4
3
2
1
0
-1
-2
-3
-4
Log(2) reads ratio L.inf Sb2000.1/WT
0
150
300
450
600
750
900
1050
1200
1350
1500
Nucleotide position (x10³)

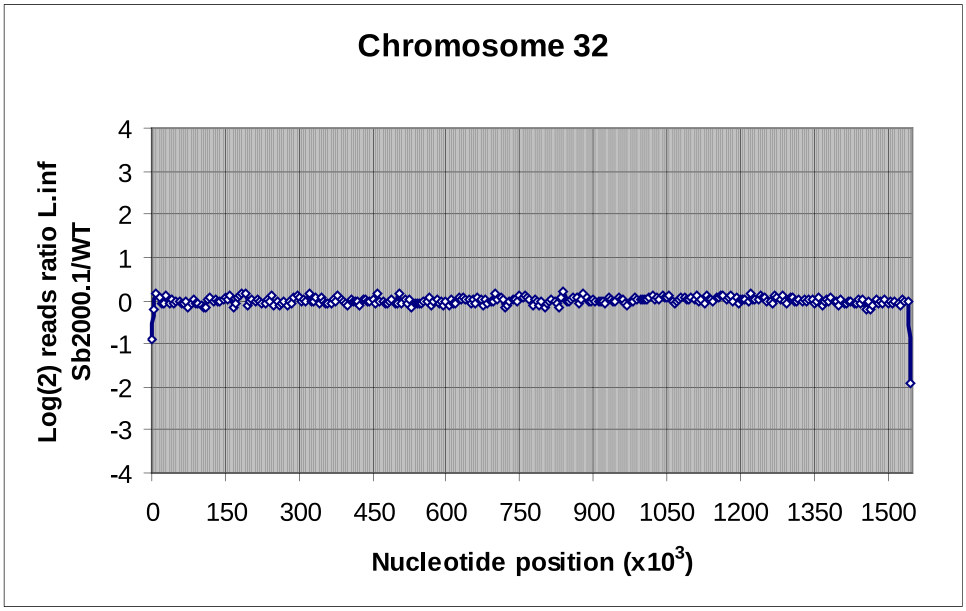
Chromosome 32
4
3
2
1
0
-1
-2
-3
-4
Log(2) reads ratio L.inf Sb2000.1/WT
0
150
300
450
600
750
900
1050
1200
1350
1500
Nucleotide position (x10³)

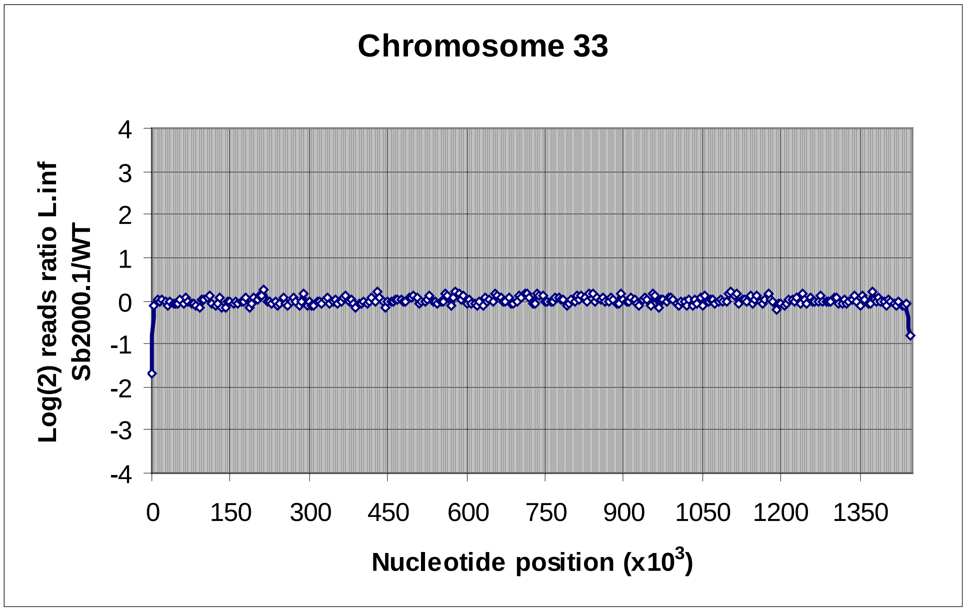
Chromosome 33
Log(2) reads ratio L.inf Sb2000.1/WT
4
3
2
1
0
-1
-2
-3
-4
0
150
300
450
600
750
900
1050
1200
1350
Nucleotide position (x10^3)

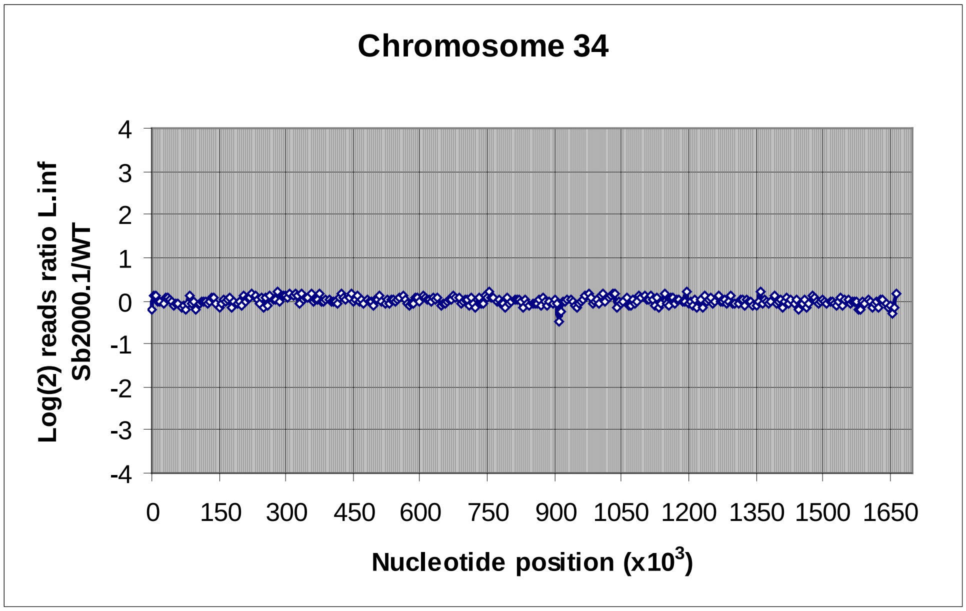
Chromosome 34
Log(2) reads ratio L.inf Sb2000.1/WT
4
3
2
1
0
-1
-2
-3
-4
0
150
300
450
600
750
900
1050
1200
1350
1500
1650
Nucleotide position (x10^3)

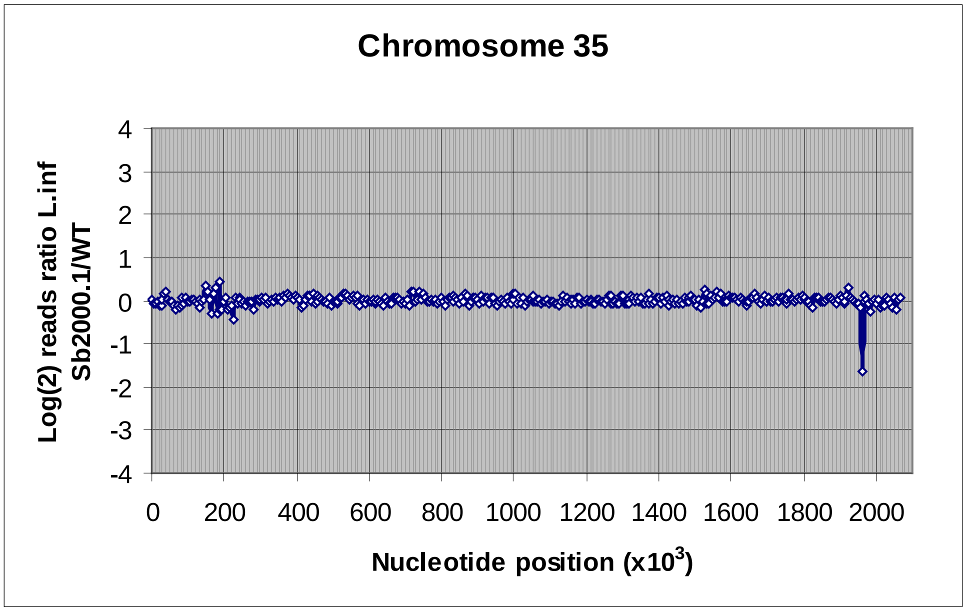
Chromosome 35
Log(2) reads ratio L.inf Sb2000.1/WT
4
3
2
1
0
-1
-2
-3
-4
0
200
400
600
800
1000
1200
1400
1600
1800
2000
Nucleotide position (x10^3)

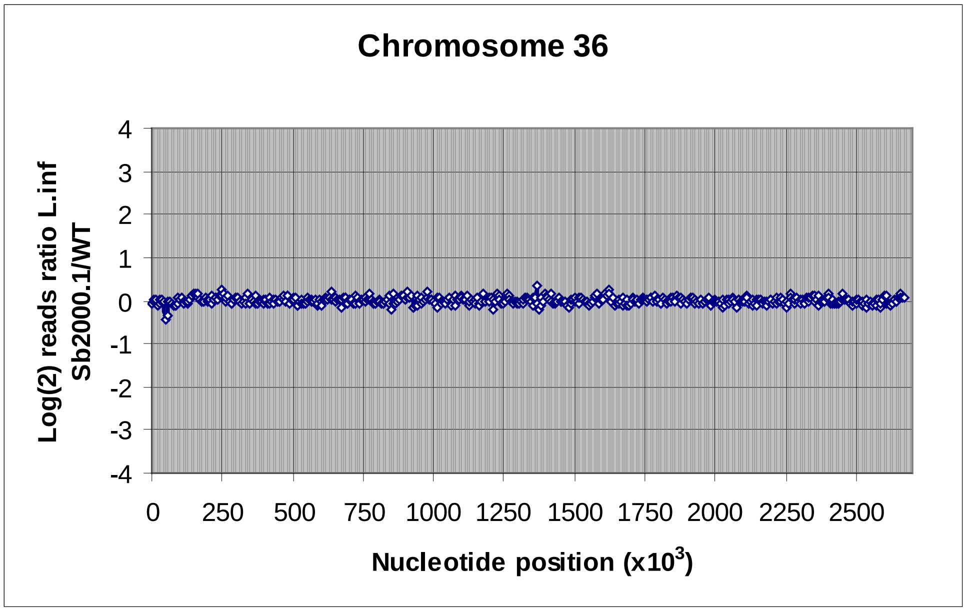
Chromosome 36
Log(2) reads ratio L.inf Sb2000.1/WT
4
3
2
1
0
-1
-2
-3
-4
0
250
500
750
1000
1250
1500
1750
2000
2250
2500
Nucleotide position (x10^3)
